# Cadmium overkill: autophagy, apoptosis and necrosis signalling in endothelial cells exposed to cadmium

**DOI:** 10.1007/s00018-015-2094-9

**Published:** 2015-11-20

**Authors:** Barbara Messner, Adrian Türkcan, Christian Ploner, Günther Laufer, David Bernhard

**Affiliations:** Cardiac Surgery Research Laboratory, Department of Surgery, Medical University of Vienna, AKH, Level 8 G9.03, Währinger Gürtel 18-20, 1090 Vienna, Austria; Plastic, Reconstructive and Aesthetic Surgery Innsbruck, Department of Operative Medicine, Innsbruck Medical University, Innsbruck, Austria; Cardiac Surgery Research Laboratory Innsbruck, University Clinic for Cardiac Surgery, Innsbruck Medical University, Innsbruck, Austria

**Keywords:** Apoptosis, Necrosis, Autophagy, p53, Lysosome, Calcium

## Abstract

Apoptosis, necrosis, or autophagy—it is the mode of cell demise that defines the response of surrounding cells and organs. In case of one of the most toxic substances known to date, cadmium (Cd), and despite a large number of studies, the mode of cell death induced is still unclear. As there exists conflicting data as to which cell death mode is induced by Cd both across various cell types and within a single one, we chose to analyse Cd-induced cell death in primary human endothelial cells by investigating all possibilities that a cell faces in undergoing cell death. Our results indicate that Cd-induced death signalling starts with the causation of DNA damage and a cytosolic calcium flux. These two events lead to an apoptosis signalling-related mitochondrial membrane depolarisation and a classical DNA damage response. Simultaneously, autophagy signalling such as ER stress and phagosome formation is initiated. Importantly, we also observed lysosomal membrane permeabilization. It is the integration of all signals that results in DNA degradation and a disruption of the plasma membrane. Our data thus suggest that Cd causes the activation of multiple death signals in parallel. The genotype (for example, p53 positive or negative) as well as other factors may determine the initiation and rate of individual death signals. Differences in the signal mix and speed may explain the differing results recorded as to the Cd-induced mode of cell death thus far. In human endothelial cells it is the sum of most if not all of these signals that determine the mode of Cd-induced cell death: programmed necrosis.

**Electronic supplementary material:**

The online version of this article (doi:10.1007/s00018-015-2094-9) contains supplementary material, which is available to authorized users.

## Introduction

Cadmium (Cd) is a toxic heavy metal and pollutant which is ubiquitously distributed in our environment. Human uptake occurs mainly by diet and exposure to cigarette smoke [1, 2]. The primary target organs in the human body are the kidneys, liver, testes, bones [1–5] and those of the cardiovascular system [6]. Furthermore, Cd is also known to be carcinogenic within different organs and tissues, which has led the International Agency for Research on Cancer to classify Cd as a Group I human carcinogen [7, 8].

Based on the variety of target organs of Cd-induced toxicity, a mass of in vitro data exists investigating the effect of Cd on different cell types. Further, Cd triggers different pathways within different cell types, rendering the landscape even more complex. In this respect one of the most useful examples is in Cd-induced cell death. In general and based upon knowledge gained thus far cell death may be classified into apoptosis, necrosis and autophagy, whereby, above all, detailed molecular and biochemical analyses are indicative of an alternative classification. In this regard, the morphological system of classification is being replaced by a functional system based on the demonstration of detailed signalling pathways and involved molecules [9]. Further adding to the complexity of signalling pathways in response to Cd toxicity, outcome is also dependent on Cd concentration; this may serve to explain some of the contradictory reports presently existing as to Cd-induced cell death. Interestingly, a review of literature revealed that even within one cell type different pathway outcomes are described. Important examples of contradictory findings regarding the Cd-induced cell death signals and final outcome are found in experiments performed upon kidney cells. Different in vitro studies have revealed highly diverse signalling pathways, ranging from caspase-independent [10] to caspase-dependent [11–14] apoptosis, although nearly all apoptotic cell death signals have in common an attendant impairment of the mitochondria. Additionally, several studies performed upon kidney cells have demonstrated autophagic signals [15], as well as signals for apoptosis and necrosis [16–18] or apoptosis and autophagy [19, 20] within the same cell type. Similarly, Lemaire et al. has reported caspase independent Cd-induced cell death in liver cells [21], whereas within the same cells, Oh et al. and Lasfer et al. provided evidence of caspase-dependent apoptotic signals [22, 23].

In addition to accumulating in known tissues such as the kidney, liver and bone, increased Cd concentrations were detected in blood serum and aortic walls (up to 20 µM) [6] of young smokers [24]. Beyond that, we and others have demonstrated that Cd is a risk factor for the development of atherosclerosis leading to fatal cardiovascular outcomes [24–29]. Thus, in addition to activating the cell death machinery [30–32] Cd exerts further effects on endothelial cells. These effects include increased expression of the adhesion molecules ICAM-1 [33, 34] and VCAM-1 [35] which possibly facilitates the adhesion and trans-endothelial migration of leucocytes leading to vascular inflammation. Furthermore, Cd treatment is known to attenuate the production of the essential vascular signalling molecule nitric oxide (NO) [36–38] thereby reducing the responsiveness of the vascular wall to essential signals. In 2000, Liu et al. demonstrated the genotoxic effect of Cd in endothelial cells as a result of the production of reactive oxygen radicals (ROR). [39] Cd-caused endothelial dysfunction is triggered by defective migratory ability of endothelial cells and inhibited angiogenesis. [40, 41] Ultimately, exposure of endothelial cells to Cd once again initiates cell death signals with inconsistent findings regarding the final fatal outcome. Kim et al. and Jung et al. have postulated a caspase-dependent apoptotic signalling pathway [30, 31] whereas Wolf et al. detected evidence of cell membrane damage and therefore necrosis [32]. Recently, a study conducted by Dong et al. revealed a novel pathway, whereby Cd-induced apoptotic signals are inhibited and rather autophagy is induced [42]. The process of Cd-induced endothelial cell death becomes further complicated still, as we have now shown that Cd triggers a programmed form of necrotic cell death accomplished by the rupture of lysosomes [43].

Taken together, the results concerning Cd-induced cell death signalling in endothelial cells are inconsistent. Proceeding from the results of a previous study by our group [43] in which we analysed the effect of Cd as a new risk factor for atherosclerosis development on endothelial cells, the present study aims to provide a highly detailed description of cell death signals within Cd-exposed endothelial cells, from Cd uptake over initiated signalling pathways and involved organelles right up to cell execution.

## Materials and methods

### Cell culture

A detailed description of the isolation of endothelial cells (HUVECs) of umbilical cords is described elsewhere [44]. The isolation and analyses of the used cells is approved by the Ethics Committee of the Medical University of Vienna (EK Nr. 1183/2012). Endothelial cells were cultured on gelatine-coated (Sigma Aldrich, Austria) polysterene culture flasks (TPP, Switzerland) in a specialised endothelial cell culture medium (EGM-2, Lonza). Prior to each experiment, the medium was replaced by fresh medium containing the indicated Cd concentrations. The following inhibitors were used: 50 µM calpain-inhibitor III (MDL 28170, Sigma Aldrich, Germany), 2 mM 3-Methyladenine (3MA; M9281, Sigma Aldrich, Germany) and 50 µM 2-Aminoethyl diphenylborinate (2APB, D9754, Sigma Aldrich, Germany), 100 µM Dantrolene (550-072-M050, Eubio) and 10 µM BAPTA (BML-CA411-0025, Enzo Lifesciences).

### Comet assay

To detect DNA damage in endothelial cells after Cd treatment, a Comet-assay was performed according to the manufacturer’s instructions (Trevigen, Cat.No.: #4250-050-K).

### Generation of stable BCL-XL OE cells as well as p53 KD HUVECs

Generation of stable BCL-XL OE cells and knock-down of p53 in endothelial cells was performed as previously described [43]. Monitoring of stable p53 KD and BCL-XL OE in endothelial cells is shown in the Supplemental Material, Figure S10, S11.

### Quantification of intracellular Ca^2+^ concentration

After the treatment, cells were incubated with 1 µM FLUO 3/AM (Calbiochem, # 343242), a cell-permeable Ca^2+^ indicator that exhibits an increase in fluorescence upon Ca^2+^ binding. Increase in fluorescence intensity after Cd treatment was detected using flow cytometry analysis (FACS Canto II, BD Biosciences) and quantified as change in the intensity as compared to the control level. We used BAPTA-AM (BML-CA411-0025, Enzo Life Sciences) at a concentration of 10 µM as a sensitive intracellular Ca^2+^ chelator.

### Detection of intracellular Cd

After treatment the cells were handled as indicated by the manufacturer’s instructions (Leadmium™ Green AM Dye for intracellular Detection of Lead and Cadmium; Life Technologies, Cat.No.: 10024) and analysed by flow cytometry (FACS Canto II, BD Biosciences, Germany).

### Detection of changes in the mitochondrial membrane potential and staining of mitochondria

After the treatment, cells were enzymatically detached, washed with PBS and incubated with 5 µM JC-1 according to the manufacturer’s instructions (Molecular Probes, Austria). Analysis and quantification of changes in the fluorescence intensity, indicating depolarised mitochondria, was performed using a FACS Canto II (BD Biosciences, Germany). Tracking of the cellular distribution of mitochondria in Cd-treated cells as compared to the control cells was performed using Mitotracker^®^ Red FM and according to the manufacturer’s instructions (Life Technologies, Molecular Probes).

### Tracking of lysosome stability

Labelling of lysosomes was performed as previously described in Messner et al. [43]. Endothelial cells with or without an inhibitor and p53 KD cells were incubated with 15 µM Cd and 30 µM Cd, respectively for the indicated times.

### Western blot analyses

Whole protein extracts from Cd-treated and control cells were obtained by incubation of detached cells in a triple detergent lysis buffer (50 mM Tris-Chloride, 150 mM Sodium Chloride, 0.02 % Sodium Azide, 0.1 % SDS, 1 % Nonident P-40, 0.5 % Sodium Deoxycholate, 5 µg/ml Aprotinin, 1 µg/ml Leupeptin, 1 µg/ml Pepstatin and 1 mM ABESF). Cells were lysed by repeated freezing and thawing and subsequent sonication. After determination of protein concentration, equal amounts of proteins were separated (20 µg) on SDS–polyacrylamide gels and subsequently transferred onto a nitrocellulose membrane (Schleicher and Schuell, Germany). The primary antibody used was anti-phospho S51 eIF2 alpha (ab47769; Abcam, UK; dilution: 1:750). The secondary antibody used was goat anti-rabbit IgG HRP-conjugate (#31460, Thermo Scientific, Austria). To control loading of equal protein amounts, membranes were stained with Ponceau S and the GAPDH expression (ab9485; Abcam, UK, dilution 1:2500) was analysed using Western blotting (secondary antibody: goat anti-rabbit IgG HRP-conjugate #31460, Thermo Scientific, Austria). Quantification of bands was performed using ImageJ software (National Institute of Health) and the ratios of the band intensities (GAPDH to specific eIF2 alpha S51) were calculated.

### Quantification of cell death

For the analysis and quantification of Cd-induced cell death, different methods were used. Annexin V/PI staining served for the discrimination of apoptotic and necrotic cell death. The staining of the cells was performed as described in Bernhard et al. [44]. And the quantification was performed using a flow cytometry device (FACS Canto II, BD Biosciences, Germany). Annexin V and PI positive cells were summarised and quantified compared to corresponding controls to display all dead cells. Furthermore, the effect of Cd on the cell membrane integrity of endothelial cells was analysed by the quantification of lactate dehydrogenase (LDH) release using the LDH cytotoxicity kit II (Biovision) according to the manufacturer’s instructions. Released LDH was quantified as compared to the LDH content of the entire cell.

### Detection and quantification of cellular DNA content and PI staining

After permeabilization of cell membranes with 1 mg/ml of saponin, the DNA was stained with PI and the DNA content was then quantified by flow cytometry (FACS Canto II, BD Biosciences, Germany). Moreover, cells were cultivated in Lab Teks (BD Biosciences) and treated with 15 and 30 µM Cd, respectively for 48 h. After fixation with 4.5 % paraformaldehyde, cells were permeabilized with 0.2 % Triton X-100 and cell nuclei were stained with PI (1 mg/ml).

### Scanning electron microscopic images of Cd-treated endothelial cells

Fixation of treated cells was performed using 2.5 % glutaraldehyde. Thereafter, cells were dehydrated in a graded ethanol series (70, 90, 100, 100, 100 %, and acetone), desiccated by critical point drying (Emitech K850; Quorum Technologies LTD, West Sussex, UK), mounted, sputtered with gold–palladium (Polaron CA 508; Fisons Instruments, Mainz-Kastel, Germany) and examined with a JEOL JSM—5400 scanning electron microscope (Eching, Germany).

### Statistical analysis

Primary data were tested for a Gaussian distribution and equality of variances. Equal distributed data were subjected either to student’s *t* Test or to one-sided ANOVA. Statistical analyses were performed using IBM SPSS Statistics 20.0 (SPSS Inc. USA).

## Results

### Chelation of Cd by EGTA prevents toxicity and Cd treatment induces DNA strand breaks in endothelial cells

**Fig. 1:**
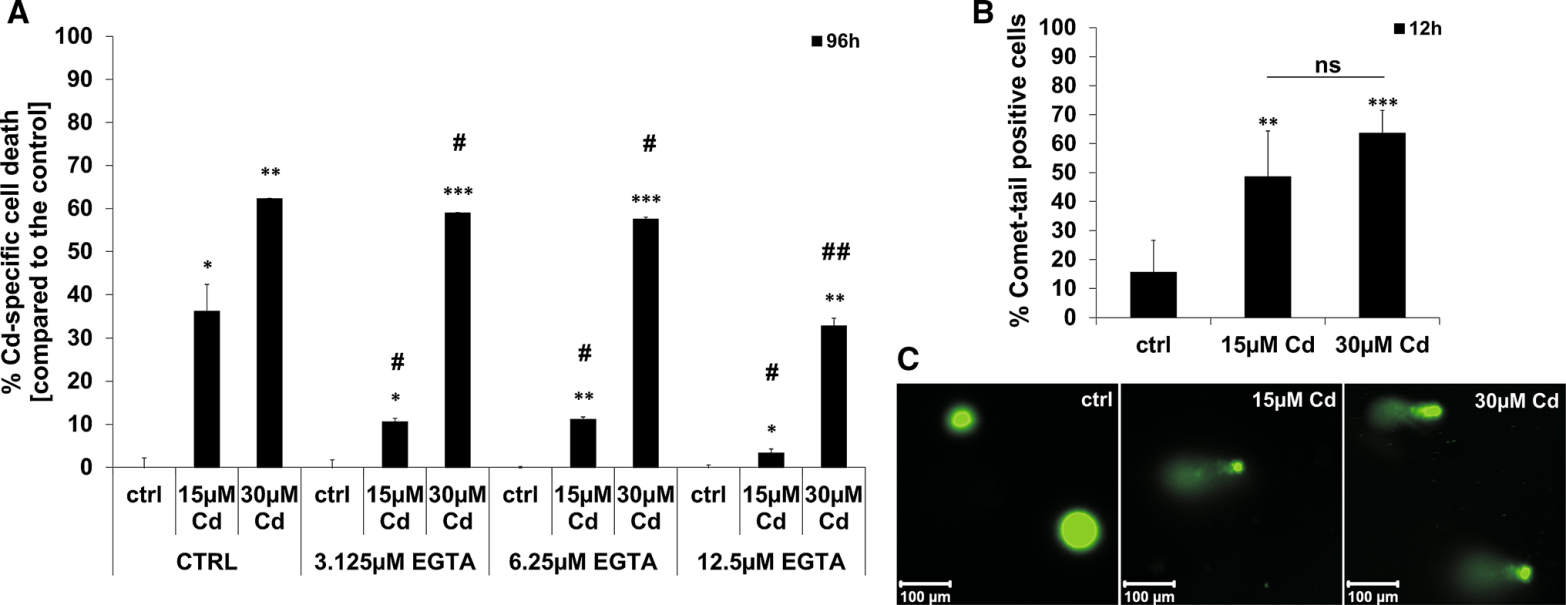
Inhibition of Cd toxicity by EGTA and the effect of Cd on endothelial DNA. **a** Shows the quantification of Cd-induced cell death (Annexin V/PI staining) after pre-treatment of cells with increasing EGTA concentrations. (**b**) Quantification of Comet-tail positive endothelial cells after treatment with 15 and 30 µM Cd for 12 h. (**c**) Representative images of cell nuclei stained with SYBR green. All experiments were performed in triplicates and were repeated at least three times. Results depict the mean ± standard deviation. *Asterisks* indicate significant differences compared to the corresponding control (*p < 0.05; **p < 0.01, ***p < 0.001) and *hash signs* indicate significant differences between the groups (^#^ *p* < 0.05; ^##^ *p* < 0.01); *ns* not significant

Pre-treatment of Cd incubated endothelial cells with the Ca^2+^ (Calcium) chelator EGTA (ethylene glycol tetra-acetic acid) significantly reduces the toxicity of this heavy metal. Quantification of flow cytometry-based Annexin V/Propidium Iodide (PI) staining revealed a significant inhibition of Cd-induced cell death by increasing EGTA concentrations after treatment with 15 or 30 µM Cd (Fig. 1a). To analyse the genotoxic effects of Cd on endothelial cells, a Comet-Assay was performed. Figure 1c shows representative images of the Comet Assay from both control and Cd-treated cells after 12 h. The amount of Comet positive cells after Cd treatment was quantified and the results are displayed in Fig. 1b. Massive DNA strand breaks are observed after treatment with 15 or 30 µM Cd. However, no influence of Cd on the cell cycle could be observed (Supplemental Material, Figure S5).

### Cd treatment provokes an increase in intracellular Ca^2+^ concentration

**Fig. 2:**
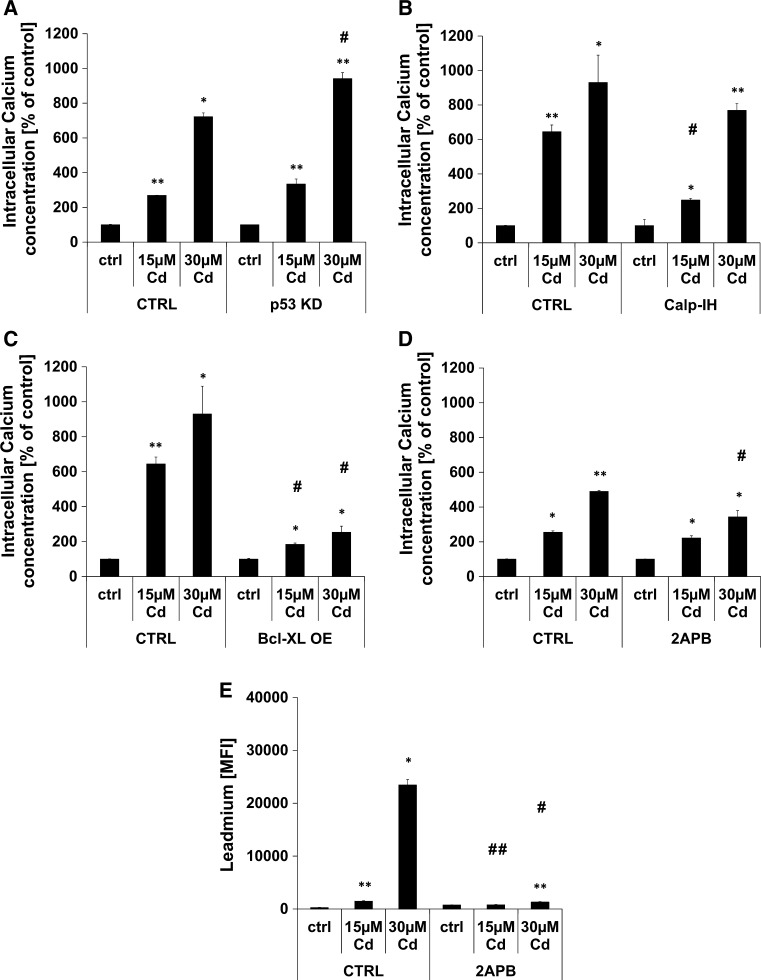
Cd induced increase in cytosolic Ca^2+^ concentration. **a**–**d** show flow cytometry-based quantifications of intracellular Ca^2+^ concentration after Cd treatment of endothelial cells for the indicated times. The effect of p53 KD (**a**), the incubation with calpain inhibitor (**b**), over-expression of BCL-XL (**c**), and of the incubation with 2APB (**d**) on cytosolic Ca^2+^ concentration is also depicted. **e** Shows flow cytometry-based analyses of the effect of 2APB on endothelial Cd uptake after 96 h. All experiments were performed in triplicates and were repeated at least three times. Results depict the mean ± standard deviation. *Asterisks* indicate significant differences compared to the corresponding (*p < 0.05; **p < 0.01) and *hash signs* indicate significant differences between the groups (^#^ *p* < 0.05; ^##^ *p* < 0.01)

In addition to the rapid genotoxic effect, Cd provokes a significant increase in intracellular Ca^2+^ concentration, already detectable after 1 h of incubation (Supplemental Material, Figure S1). Furthermore, the involvement of the Ca^2+^ sensitive non-lysosomal cysteine protease calpain was analysed by the usage of the calpain I and II inhibitor (MDL 28170) showing a significant inhibition of Ca^2+^ flux in cells treated with 15 µM Cd, but no effect in cells treated with 30 µM Cd after 24 h (Fig. 2b). To rule out the role of p53 in Cd-induced cell death as previously suggested [43], we generated p53 KD endothelial cells to address the question of whether Cd-induced DNA breaks result in a p53-dependent cell death response. Although p53 is involved in the Cd-induced cell death pathway, a knock-down of p53 was not able to inhibit the intracellular Ca^2+^ flux induced by Cd after 24 h (Fig. 2a).

Involvement of BCL-XL in Cd-induced Ca^2+^ flux was analysed using endothelial cells overexpressing this anti-apoptotic protein, and Fig. 2c shows that this overexpression is highly efficient in inhibiting Ca^2+^ flux. To test the hypothesis that Cd treatment induces a substantial release of Ca^2+^ from the endoplasmatic reticulum (ER), 2APB (2-Aminoethoxydiphenyl borate) was used to inhibit InsP_3_ (Inositol 1,4,5-triphosphate)-mediated Ca^2+^ release. Flow cytometry-based analyses uncovered that 2APB incubation is not able to inhibit Cd-induced Ca^2+^ flux entirely, but does so significantly as observed 24 h after treatment with 30 µM (Fig. 2d). Referring to the controversially discussed specificity of 2APB as restricted to ER Ca^2+^ pumps, we analysed the effect of this inhibitor upon the cellular Cd uptake. Leadmium-dye-based intracellular Cd quantification indicates that 2APB significantly inhibits Cd uptake (Fig. 2e).

### Cd treatment induces mitochondrial membrane depolarisation and changes in the mitochondrial distribution

**Fig. 3:**
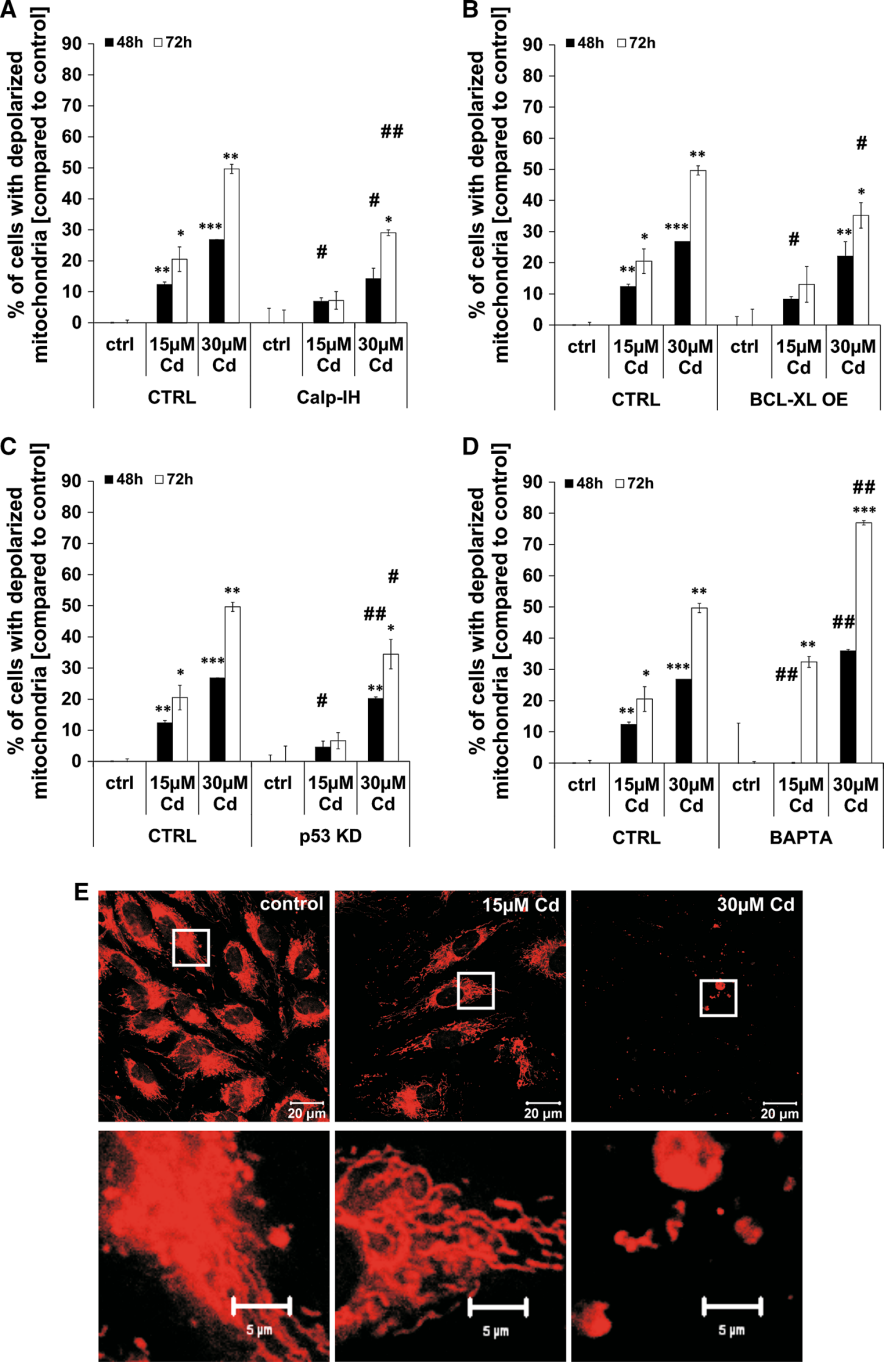
Cd impairs mitochondrial membrane potential and reduces mitochondrial mass. Flow cytometry-based analyses of mitochondrial membrane potential in Cd-treated endothelial cells are shown in (**a**–**d**). Cd-treated endothelial cells were incubated either with the calpain inhibitor or BAPTA and mitochondrial membrane potential was assayed using a JC-1 assay and quantified by flow cytometry analyses (**a**, **d**). **b**, **c** The effects of either p53 KD or BCL-XL OE on mitochondrial membrane potential in Cd-treated endothelial cells. **e** The first row shows representative images of Cd-treated endothelial cells (48 h) stained with MitoTracker Red and the *second row* shows magnifications indicated by the corresponding *white boxes*. All experiments were performed in triplicates and were repeated at least three times. Results depict the mean ± standard deviation. *Asterisks* indicate significant differences compared to the corresponding (*p < 0.05; **p < 0.01, ***p < 0.001) and *hash signs* indicate significant differences between the groups (^#^ *p* < 0.05; ^##^ *p* < 0.01)

Further in-depth characterisation of Cd-induced cell death signals uncovered an involvement of the mitochondria. Treatment of endothelial cells with 15 µM Cd as well as 30 µM Cd provokes a significant depolarisation of the mitochondria over an incubation period of 72 h (Fig. 3). As calpains are also present in the mitochondria, the effect of the calpain inhibitor on mitochondrial depolarisation was tested. Figure 3a shows that the calpain inhibitor was efficient in inhibiting the depolarisation after treatment with 15 and 30 µM Cd over a period of 72 h. A stable overexpression of the anti-apoptotic protein BCL-XL was able to inhibit mitochondrial depolarisation although the effect is less pronounced as compared to the effect of the calpain inhibitor (Fig. 3b). A similar inhibitory effect on mitochondrial depolarisation was detected in cells with a stable p53 knock-down (KD) (Fig. 3c). Furthermore, chelation of Ca^2+^ by the chelating agent BAPTA completely inhibits mitochondrial depolarisation induced by 15 µM Cd after 48 h, but showed no effect after treatment with 30 µM Cd or after a longer Cd exposure (up to 72 h). However, this effect can be explained by the cell-toxic effect of BAPTA treatment itself (Fig. 3d). A detailed analysis of mitochondrial structure revealed severe Cd-dependent effects on mitochondrial mass (Fig. 3e, 15 µM) and integrity (Fig. 3e, 30 µM). This strongly suggests that Cd-induced cell death is mediated by mitochondria integrity.

### Lysosomal membrane permeabilization and induction of autophagy signals are characteristic of death signalling in Cd-treated endothelial cells

**Fig. 4:**
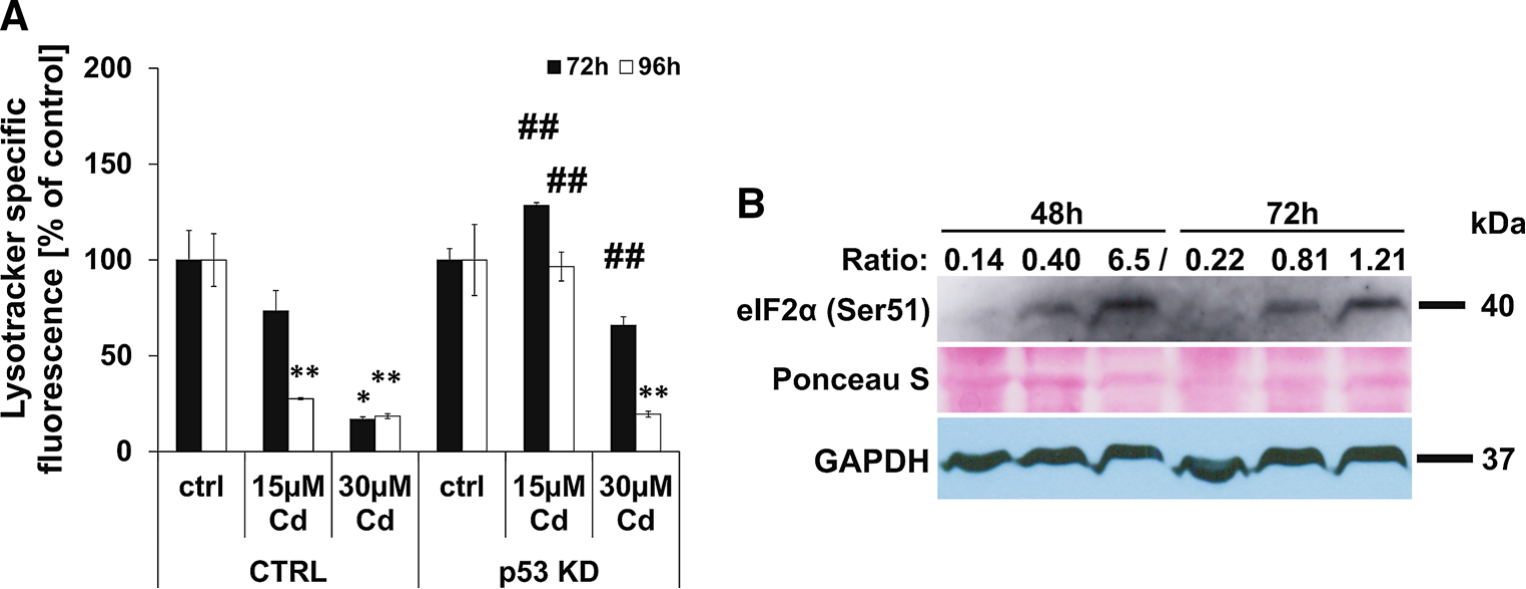
Cd-induced lysosomal membrane permeabilization and eIF2 alpha phosphorylation. Flow cytometry-based quantification of LMP was performed in endothelial cells treated with 15 and 30 µM Cd for 72–96 h. The effect of the p53 KD on Cd-induced LMP is depicted in (**a**). Western Blot-based analyses of eIF2 alpha phosphorylation in endothelial cells after Cd treatment is depicted (**b**). To compensate unequal protein loading, the Ponceau staining of membranes as well as the GAPDH expression is shown and the ratio between specific signal intensity and protein loading (GAPDH) was calculated and depicted. All experiments were performed in triplicates and were repeated at least three times. Results depict the mean ± standard deviation. *Asterisks* indicate significant differences compared to the corresponding control (*p < 0.05; **p < 0.01) and *hash signs* indicate significant differences between the groups (^##^ *p* < 0.01)

As already reported by our group, Cd treatment of endothelial cells induces autophagy signals (namely, an increase in LC3 I to LC3 II ratio). As the lysosomes are involved in autophagy signalling, we analysed stability of these organelles using flow cytometry techniques which show a strong Cd-dependent disruption of lysosome integrity (Fig. 4). Moreover, Fig. 4a shows that the stable knock-down of p53 is able to completely inhibit lysosomal degradation after treatment with 15 µM Cd over a time period of 96 h as well as in cells incubated with 30 µM Cd over 72 h. In contrast, inhibition of calpain protease or autophagy (3MA) failed to exert lysosome-protective characteristics (Supplemenatl Material, Figure S6 A, B). In response to the unfolded protein response (UPR) (also a part of autophagy signalling) within the ER, the function of eukaryotic initiation factor eIF2 alpha is inhibited by phosphorylation. Western blot-based analyses showed a phosphorylation of eIF2 alpha in response to Cd treatment (Fig. 4b).

### Classical AnnexinV/PI based flow cytometry analyses reveal apoptosis as the prevailing mode of Cd-induced cell death

**Fig. 5:**
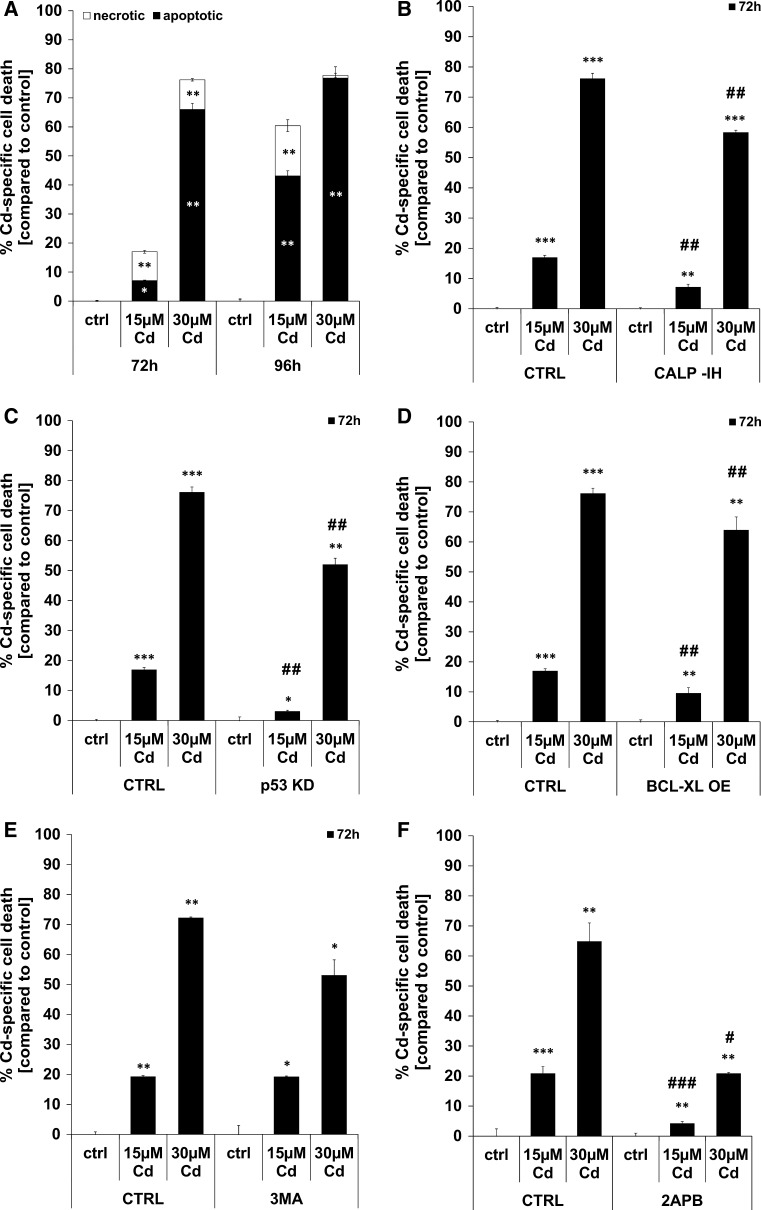
Cd treatment results in apoptotic as well as necrotic cell death in endothelial cells. Endothelial cells were treated with Cd as indicated for 72 and 96 h and the amount of necrotic as well as apoptotic cells were quantified using Annexin V/PI stainings and flow cytometry-based analyses (**a**). The cell death inhibitory effect of the calpain inhibitor (**b**), p53 KD (**c**), BCL-XL OE (**d**), autophagy inhibition (**e**) and of 2APB (**f**) is also depicted in this figure. All experiments were performed in triplicates and were repeated at least three times. Results depict the mean ± standard deviation. *Asterisks* indicate significant differences compared to the corresponding control (*p < 0.05; **p < 0.01, ***p < 0.001) and *hash signs* indicate significant differences between the groups (^#^ *p* < 0.05; ^##^ *p* < 0.01; ^###^ *p* < 0.001)

Flow cytometry-based analyses uncovered apoptosis as the prevailing cell death mode. After 72 h of treatment with 15 µM Cd, nearly equal numbers of cells are dying by apoptosis and necrosis. Treatment with a higher Cd concentration of 30 µM induces a marked increase in the amount of apoptotic cells. Prolonged treatment with 15 µM Cd over a time period of 96 h enhances necrotic and, to a greater extent, apoptotic cell death (Fig. 5a). Figure 5b depicts the calpain inhibitor’s ability to reduce the amount of dead cells after treatment with 15 and 30 µM Cd for 72 h. Likewise, the stable knock-down of p53 is able to prevent Cd-induced death over 72 h, where the effect of the knock-down is stronger than the effect of the calpain inhibitor (Fig. 5c). Overexpression of the anti-apoptotic protein BCL-XL has only a minor inhibiting effect upon cell death, yet the effect is nonetheless statistically significant at both concentrations (Fig. 5d). Neither inhibition of autophagy with 3MA (Fig. 5e) nor energy supplementation (Supplemental Material, Figure S9 A–C) protects cells treated with 15 µM Cd. However, we observed an almost-significant increase of viability in 30 µM Cd-treated cells (*p* = 0.079; Fig. 5e). Based on the controversial results obtained with 2APB (partial inhibition of Ca^2+^ flux and inhibition of Cd uptake), the effect of this inhibitor on Cd-induced cell death was assessed. 2APB was able to completely inhibit death of cells treated with 15 µM Cd and showed the most potent cell death inhibitory effect in cells treated with 30 µM Cd as compared to all other inhibitors, knock-downs and over-expressions (Fig. 5f).

### DNA degradation is characteristic of end-stage processes of Cd-induced endothelial cell death

**Fig. 6:**
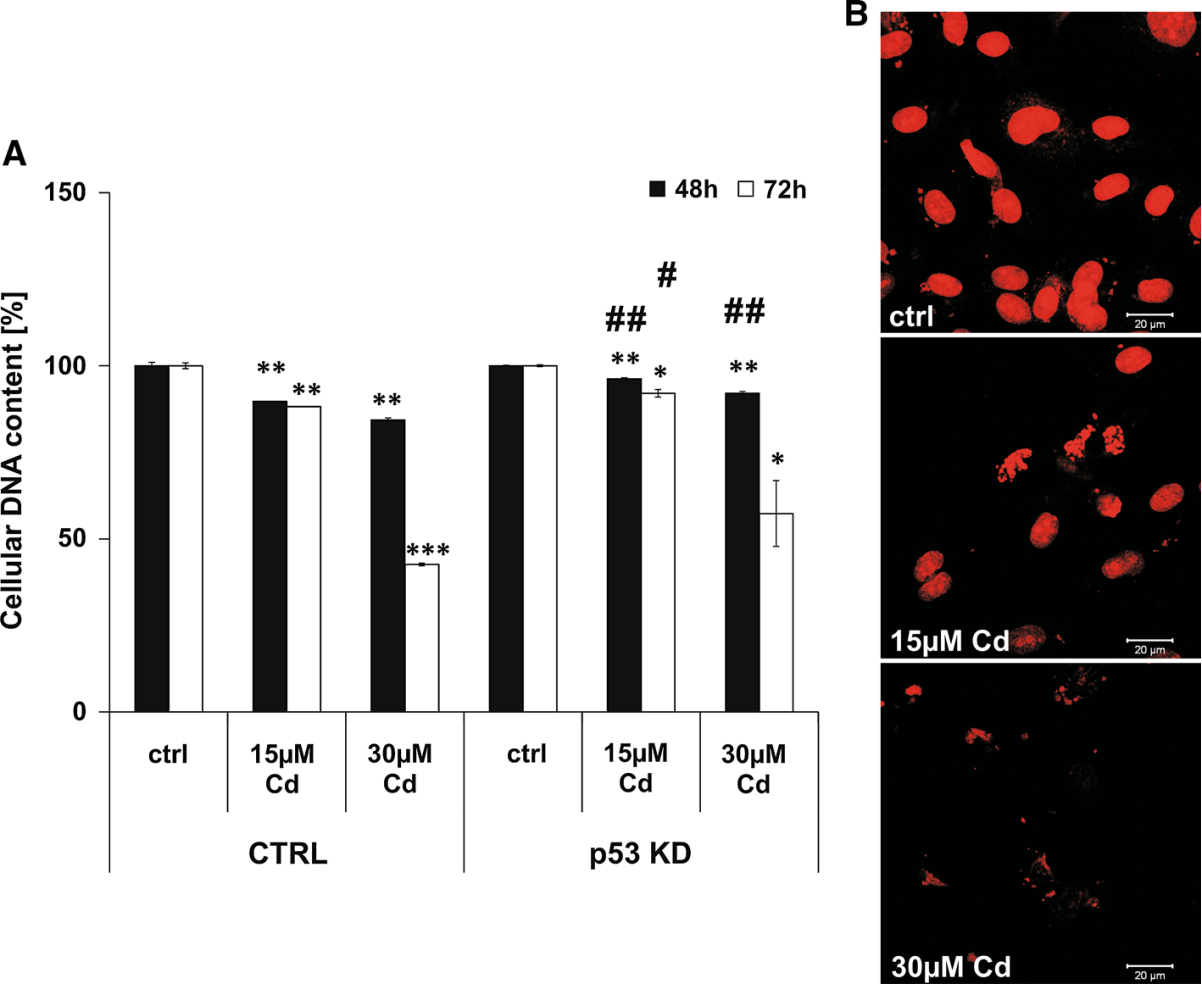
Cd treatment of endothelial cells provokes complete DNA degradation. Endothelial cells were treated with 15 and 30 µM Cd for 48 and 72 h and the cellular DNA content was quantified in permeabilized cells using PI staining and flow cytometry-based analyses. The effect of p53 KD on Cd-induced DNA degradation is shown in (**a**). **b** Representative images of the PI stained endothelial nuclei after Cd treatment for 48 h. All experiments were performed in triplicates and were repeated at least three times. Results depict the mean ± standard deviation. *Asterisks* indicate significant differences compared to the corresponding control (*p < 0.05; **p < 0.01, ***p < 0.001) and *hash signs* indicate significant differences between the groups (^#^ *p* < 0.05; ^##^ *p* < 0.01)

In contrast to the flow cytometry-based analyses of Annexin V/PI stainings, measurement of DNA content in Cd-treated cells suggests a necrotic outcome (Fig. 6). Inhibition of Cd-induced DNA degradation was achieved by the stable knock-down of p53 as these cells were unaffected when incubated with 15 µM Cd after 48 and 72 h. Inhibition of DNA degradation by p53 KD was also visible after treatment with 30 µM Cd, despite the effect only being significant after 48 h (with a non-significant trend observed after 72 and 96 h; Fig. 6a). Overall, no inhibitory effect on Cd-induced DNA degradation could be observed after the pre-incubation with the calpain inhibitor and the autophagy inhibitor (Supplemental Material, Figure S7 A, B). Moreover, the pre-incubation of Cd-treated cells with the Ca^2+^ -Mg^2+^ -dependent endonuclease inhibitor Aurintricarboxylic acid (ATA) does not abolish the progressive DNA degradation (Supplemental Material, Figure S8A). However, incubation with the extracellular Ca^2+^ chelator EGTA prevents Cd-induced DNA degradation completely (Supplemental Material, Figure S8B). Figure 6b depicts images of stained cell nuclei of control and Cd-treated cells after 48 h of incubation. Control cells show intact cell nuclei containing DNA, whereas the nuclei of Cd-treated cells show reduced DNA content to almost complete degradation.

### Cd induces the permeabilization of the plasma membrane and release of LDH

**Fig. 7:**
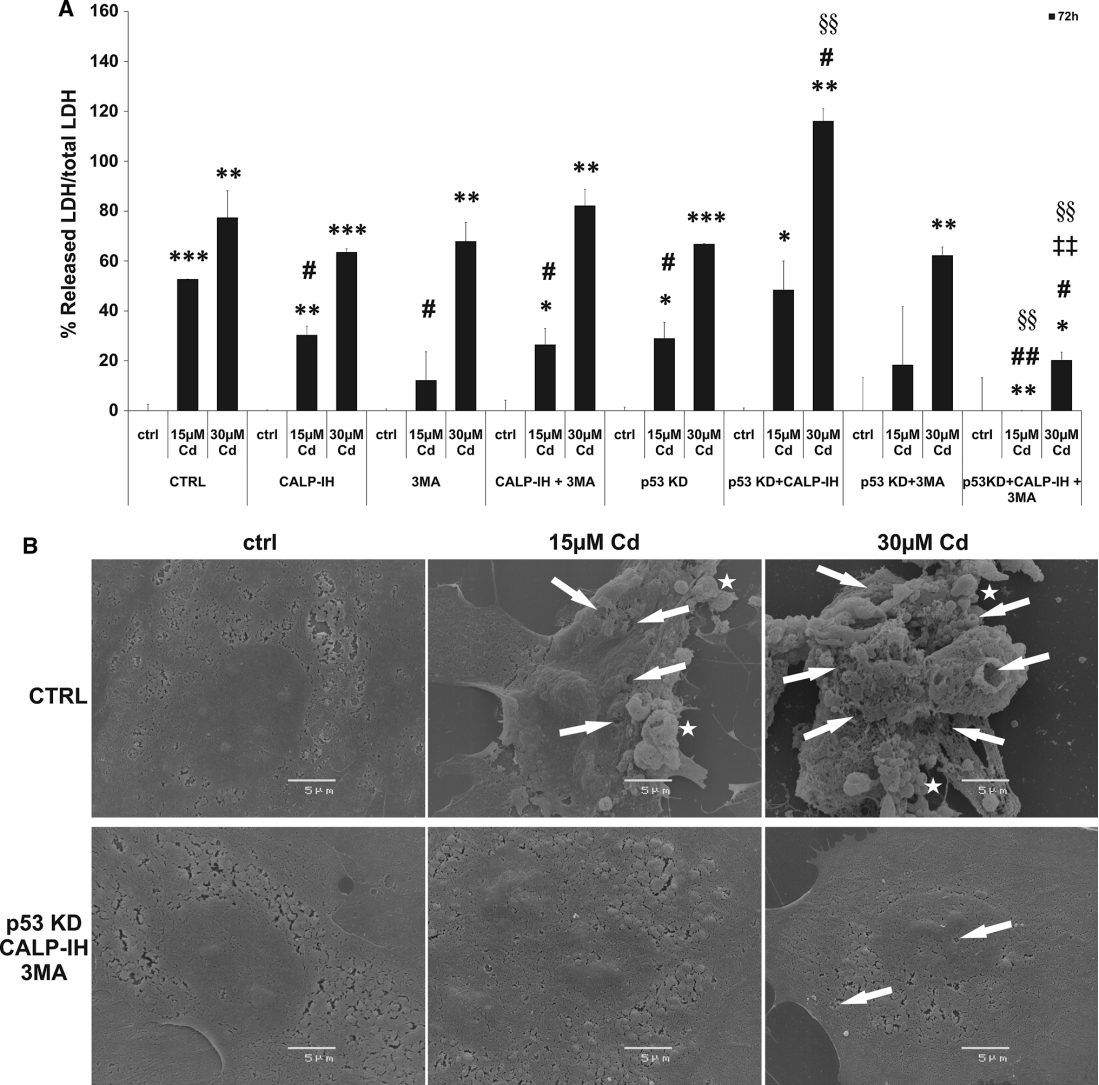
Cd induced membrane permeabilization and LDH release. **a** The analyses of Cd-induced LDH release from endothelial cells into the cell culture supernatant as well as the effects of inhibitors (calpain inhibitor and 3MA) and p53 KD and also their combined effect. Representative scanning electron microscopic images of wild-type endothelial cells treated with Cd and p53 KD cells pre-incubated with the calpain inhibitor and 3MA treated with Cd are shown (*stars* indicate membrane blebs and *arrows* mark holes in the plasma membrane) (**b**). All experiments were performed in triplicates and were repeated at least three times. Results depict the mean ± standard deviation. *Asterisks* indicate significant differences compared to the corresponding control (ctrl; *p < 0.05; **p < 0.01, ***p < 0.001), *hash signs* indicate significant differences compared to the control group without the inhibitor or KD (CTRL; ^#^ *p* < 0.05; ^##^ *p* < 0.01; ^###^ *p* < 0.001) and *section signs* indicate significant differences as between the p53 cells without the inhibitor and p53KD cells with the inhibitor (^§§^ *p* < 0.01)

Further confirmation of necrosis as the final outcome of Cd-induced endothelial cell death is the permeabilization of the plasma membrane, shown by a massive LDH release and through scanning electron microscopic-images (Fig. 7). LDH release induced by 15 µM Cd could be significantly suppressed by inhibition of calpain activity, 3MA incubation, p53 KD and the combination of the calpain inhibitor with 3MA. LDH release induced by 30 µM Cd is inhibited neither by the previously mentioned inhibitors nor by a knock-down of p53. Furthermore, a combination of p53 KD and the individual inhibitors was not effective in inhibiting membrane permeabilization. However, the combined treatment of p53 KD endothelial cells with the calpain inhibitor and 3MA completely abolishes LDH release in cells treated with 15 µM Cd and reduces the amount of LDH released from cells treated with 30 µM Cd to below 21 % of the control level (Fig. 7a). To illustrate the Cd-induced effects on the endothelial cell surface, scanning electron microscopic images were taken and are depicted in Fig. 7b. Shown are wild-type endothelial cells (CTRL) treated with Cd and p53 KD cells incubated with the calpain inhibitor and 3MA. Untreated cells show a flattened shape with an intact plasma membrane and the location of the nucleus is clearly visible. CTRL cells treated with 15 µM Cd exhibit blebs of different size on their surface (labelled with stars) and in addition several leaks in the plasma membrane (labelled with arrows). Moreover, shrinkage of these cells is observable. In contrast, p53 KD cells incubated with the calpain inhibitor and 3MA, and treated with 15 µM Cd are completely intact. Wild-type cells incubated with 30 µM Cd exhibit a large number of blebs of different sizes (labelled with stars) and the plasma membrane appears to be completely disintegrated (labelled with stars). As expected from the results of the LDH assay, p53 KD cells incubated with the calpain inhibitor and 3MA and treated with 30 µM Cd showed a slight detachment from the surface and only a few small holes in the membrane (Fig. 7b).

## Discussion

The chelating agents EGTA and EDTA (ethylenediamineteteaacetic acid) which are clinically applied for the treatment of metal intoxications clearly reduce Cd-induced DNA degradation in a concentration-dependent manner by forming extracellular complexes with the metal ion, thereby hindering the cellular uptake of Cd. [45] Once taken up by cells, Cd is known to cause DNA damage as already proven in different cell types such as liver cells [46, 47], fibroblasts [48], lung cells [49], and epithelial cells [50]. Similarly, endothelial cells are also sensitive to Cd-induced DNA damage as demonstrated by our previous data showing an increase in the expression of the DNA damage response protein p53 [29] and the latest data showing a pronounced amount of Comet-positive cells after 12 h of Cd treatment. Interestingly, the extent of damage is comparable between 15 and 30 µM Cd, although the number of dead cells is significantly higher after treatment with 30 µM Cd. Furthermore, the involvement of DNA damage and p53 in Cd-induced cell death signalling is shown by the inhibition of cell death (and the preservation of proliferation after Cd treatment, Supplemental Material, Figure S3 A, B) after the knock-down of p53. This demonstrates that Cd causes DNA damage in endothelial cells even at lower concentrations leading to cell death with the involvement of p53.

In addition to the induction of DNA damage, Cd treatment of endothelial cells induces a rapid and substantial increase in intracellular Ca^2+^ concentration within 6 h. Ca^2+^, as a universal intracellular messenger, influences a wide range of cellular processes (such as cell death signalling) when the concentration is elevated in the cytosolic compartment [51, 52]. Cytosolic Ca^2+^ increase is accomplished by either uptake from the extracellular space or release from intracellular Ca^2+^ stores such as the ER and the mitochondria. 2APB, a critically discussed blocker of Ca^2+^ release from the ER, reduces Cd-induced cytosolic Ca^2+^ concentration but simultaneously inhibits both cellular Cd uptake and Cd-induced cell death significantly. Moreover, chelation of extracellular Ca^2+^ by EGTA protects endothelial cells from Cd-induced cell death. Ca^2+^ concentration showed no link to p53-dependent DNA damage signalling. Another indication of the release of Ca^2+^ from the ER through Cd treatment is the fact that the over-expression of BCL-XL—an anti-apoptotic BCL-2 family member located in the ER membrane—is able to inhibit the increase of cytosolic Ca^2+^ concentration in endothelial cells. However, BCL-XL OE did not effectively prevent endothelial cell death following Cd incubation. Withe and Li et al. confirmed this hypothesis by providing evidence of a regulation of the inositol trisphosphate receptor (InsP_3_R) by BCL-XL through direct interaction to increase apoptosis resistance [53]. Transmission of the cell death signal from Ca^2+^ is mediated by a family of Ca^2+^-dependent non-lysosomal cysteine proteases known as calpains [54]. Interestingly, inhibition of calpain activity reduces intracellular Ca^2+^ concentration significantly at low Cd concentrations. Although an involvement of autophagy in Cd-induced endothelial cell death is already proven [43], no interplay between increased cytosolic Ca^2+^ and autophagy could be detected.

The complex signalling pathway is extended by an involvement of the mitochondria and a variety of factors influencing mitochondrial function [29]. Depolarisation of mitochondria as well as the simultaneous loss of mitochondrial mass certainly occurs late in the Cd-induced cell death process (after 48 h). Similarly to p53-mediated cell death in neuronal cells [55], Cd toxicity leads to mitochondrial depolarisation through p53, which was reduced by the p53 KD. Since outer membrane permeabilization is weakly inhibited by BCL-XL OE, Bax activation is arguably involved in mitochondrial depolarisation. Additionally, Cd-induced mitochondrial depolarisation is also indirectly induced by calpain activation (as already shown in endothelial cells [55]) as well as directly through elevated Ca^2+^ levels as indicated by the limited inhibitory effect of intracellular Ca^2+^ chelation. Lastly, the close proximity between ER cisternae and the mitochondria allows for local Ca^2+^ oscillation into mitochondria [56]. All these factors ultimately lead to a central element in apoptosis, which is signal transduction through caspases, known as apoptosis executors. In the case of Cd-treated endothelial cells we have previously shown that caspase-3 is cleaved, but Cd inhibits its activity [29]. Therefore, the apoptotic signalling is halted at this point, underpinned by unchanged intracellular ATP concentration (Supplemental Material, Figure S2).

Ca^2+^ imbalance and calpain activation not only lead to mitochondrial signalling but also result in the initiation of the UPR and induce ER stress, indicated by the phosphorylation of eIF2 alpha. Phosphorylation of eIF2 alpha disables initiation of translation and thereby inhibits the production of novel unfolded proteins to reduce ER stress. If this protective feedback loop fails, apoptotic signalling is induced—likely, once again, via the mitochondria [57]. Importantly, the PERK-eIF2 alpha complex is responsible for the up-regulation of Atg12 and thereby induces autophagosome formation. The induction of autophagy signals by Cd treatment, shown by an increased LC3 II/LC3 I ratio [43] as well as cell death protection through 3MA incubation, might therefore be triggered by UPR and eIF2 alpha phosphorylation. However, autophagy is inhibited since Cd treatment of endothelial cells provokes lysosomal membrane permeabilization (LMP) [43].

Lysosomes are known as “suicide bags” indicating their role in both cell death mainly through necrosis but also in survival mechanisms in the case of autophagy [58]. As previously described, Cd-induced LMP provokes the release of nucleases triggering complete DNA degradation, as well as proteases and lipases causing permeabilization of the plasma membrane [43]. Previously, BCL-XL OE demonstrated a stabilizing effect on LMP which suggests the involvement of the mitochondria [43]. An explanation of this effect could be the fact that BCL-XL attenuates BAX activity, a pro-apoptotic protein which is known to induce LMP. Moreover, as BCL-XL is also present in the ER-membrane, an involvement of this organelle in LMP cannot be excluded. Lastly, we were able to provide a connection between a DNA damage-induced increased expression of p53 and LMP, as the knock-down of p53 rescues cells from Cd-induced LMP, which is already shown in myeloid leukaemic cells [59]. The signalling cascade from increased p53 protein levels to LMP is further confirmed by the protection of DNA degradation and the inhibition of plasma membrane rupture by p53 KD. In contrast to data from neuronal cells by Villalpando Rodriguez et al. [60], in endothelial cells no connection between calpain activation, LMP induction and DNA degradation could be observed. However, an inhibition of calpain activity inhibits cell membrane permeabilization and preserves proliferation (Supplemental Material, Figure S4 A, B), possibly through an as yet unknown mechanism. Comparable to the inhibition of calpain activity and the knock-down of p53, the disruption of autophagy signals also inhibits plasma membrane permeabilization. Remarkably, an inhibition of apoptotic signalling (by p53 KD and inhibition of calpain activity) combined with autophagy (by using 3MA) was able to completely prevent necrotic cell death induced by 15 µM Cd, and to significantly reduce the number of necrotic cell deaths induced by 30 µM Cd.

**Fig. 8:**
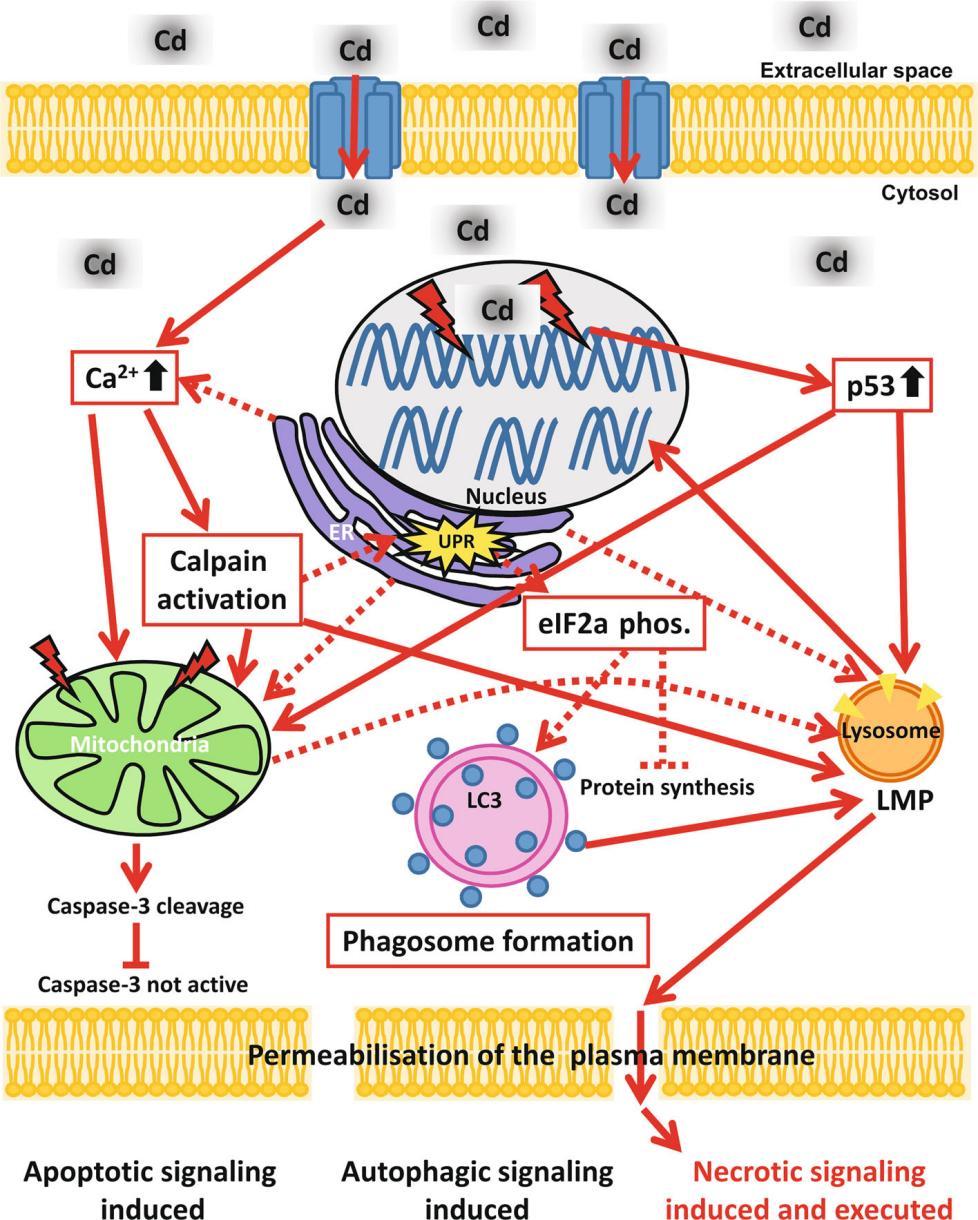
Schematic representation of Cd-induced signalling pathways in endothelial cells. After uptake of Cd in endothelial cells [possibly through divalent metal transporter 1 (DMT-1)] the cytosolic Ca^2+^ concentration rises. Besides being taken up from the extracellular space, Ca^2+^ could also originate from the ER. Redirection of the signal to the mitochondria could take place directly by Ca^2+^ or also indirectly by the activation of calpains. Forwarding of the apoptotic signalling from the mitochondria occurs via the cleavage of caspase-3, but the apoptotic signal is halted at this point as Cd treatment is known to simultaneously inhibit caspase-3 activity. Alongside affecting the mitochondria, Cd treatment impairs also the ER function and induces ER stress (shown by UPR). Cd-induced ER stress may stop protein synthesis via eIF2 alpha phosphorylation and also induce autophagic signalling, potentially as a survival mechanism. An additional target of Cd-induced toxicity in endothelial cells is cellular DNA. Cd-induced DNA damage results in upregulation of the DNA damage response protein p53, which in turn is shown to influence mitochondrial membrane potential. The central organelle in Cd-induced toxicity is the lysosome, as Cd-induced death signals from the mitochondria, the ER and p53 are finally “merging” at the lysosome, inducing LMP. LMP induction is in consequence responsible for complete DNA degradation and also for plasma membrane permeabilization and therefore ultimately for the execution of necrosis in Cd-treated endothelial cells although apoptotic as well as autophagy signals are triggered as well

In conclusion, although necrosis is commonly known as an accidental non-regulated death process, Cd-treated endothelial cells present a highly regulated and complex cell death signalling mechanism which involves a variety of different cell organelles. Apoptotic and autophagy signals induced through Cd toxicity culminate in necrosis (Fig. 8) and can be prevented by the inhibition of all activated signalling pathways. In summary, our work supports the growing body of evidence of the existence of a highly regulated form of necrosis, as recently reviewed by Vanden Berghe et al. [61].

### Study limitations

The present study is based on several hypotheses, which limit the extensibility of the observed results to the situation in humans. A central limitation is that all observations made are based on an in vitro cell culture model including the chosen Cd concentrations, which are not necessarily applicable to the situation in humans. Cd-concentrations chosen are based on studies by Abu-Hayyeh et al. [6] who reported on the occurrence of up to 20 µM of Cd in the aortic wall of chronic smokers, and a study by Bergström et al. reporting up to 50 fold increased Cd concentrations in the vascular intima compared to the blood stream. [62].

## Electronic supplementary material

Supplementary material 1 (PDF 1353 kb)

## Abbreviations

Cd: Cadmium
HUVEC: Human umbilical vein cells
ICAM-1: Intracellular adhesion molecule-1
VCAM-1: Vascular cell adhesion molecule-1
NO: Nitric oxide
ROR: Reactive oxygen radicals
Ca^2+^: Calcium
PI: Propidium iodide
EGTA: Ethylene glycol tetra-acetic acid
EDTA: Ethylenediaminetetraacetic acid
ER: Endoplasmatic reticulum
2APB: 2-Aminoethoxydiphenyl borate
KD: Knock down
OE: Over-expression
3MA: 3-Methyladenine
LDH: Lactate dehydrogenase
LMP: Lysosomal membrane permeabilization
