# Supplementary material for: Cadmium overkill: autophagy, apoptosis and necrosis signalling in endothelial cells exposed to cadmium
Source: Cell Mol Life Sci. 2015 Nov 20;73(8):1699–713. doi: 10.1007/s00018-015-2094-9 (PMC4805700; doi:10.1007/s00018-015-2094-9)
Supplement: Supplementary file 1 — Supplementary material 1 (PDF 1353 kb) [file 18_2015_2094_MOESM1_ESM.pdf]

SUPPLEMENTAL MATERIAL

to

Messner et al.

Cadmium Overkill: Autophagy, Apoptosis and Necrosis Signalling in Endothelial Cells Exposed to Cadmium

Running Title: Complexity of Cd Induced Death Signalling

Barbara Messner [1], Adrian Türkcan [1], Christian Ploner [2], Günther Laufer [1], and David Bernhard [3]

MATERIAL AND METHODS

Quantification of cell death

For the analysis and quantification of Cd-induced cell death, Annexin V/Propidium iodide (PI) staining was used to determine the amount of dead cells (apoptotic and necrotic). Endothelial cells were seeded in 6-well plates and pre- and co-incubation with indicated Sucrose, Fructose, and Mannitol concentrations and treated with 15µM and 30µM Cd respectively, for the indicated times. The staining of the cells was performed as described in Bernhard et al. (Bernhard et al. 2003) and the quantification was performed using a flow cytometry device (FACS Canto II, BD Biosciences, Germany). Annexin V and PI positive cells were summarized and quantified compared to corresponding controls to display all dead cells.

Quantification of intracellular Ca2+ concentration

For the analysis and quantification of intracellular Ca^{2+} concentrations, HUVECs were seeded in 6-well plates and incubated with 15µM and 30µM Cd respectively for the indicated times. After the treatment, cells were incubated with 1µM FLUO 3/AM, a cell-permeable Ca^{2+} indicator that exhibits an increase in fluorescence upon Ca^{2+} binding. Increase in fluorescence intensity after Cd-treatment was detected using flow cytometry analysis (FACS Canto II, BD Biosciences) and quantified as change in the intensity compared to the control.

ATP-Assay

Intracellular ATP-concentration was measured using the ApoSENSOR Viability Kit from Biovision, according to the manufacturer's instructions (Biovision, Cat.No.: K254-200). ATP-concentration was analysed as ATP amount per number of cells.

Quantification of the number of viable cells

To analyse the number of cells, endothelial cells were treated with 15µM and 30µM Cd respectively over a time period of 96h. Quantification of the number of cells was performed using the XTT assay (Biomol, Hamburg, Germany) according to the manufacturer's instructions. As indicated, cells were pre-incubated with 50µM calpain inhibitor for 30 min before Cd treatment.

Cell cycle analyses

To analyse the influence of Cd on the cell cycle, endothelial cells were treated with 15µM and 30µM Cd respectively for 6h. After harvesting, the cells were fixed in 70% ethanol for 1h at 4°C. Afterwards, cells were washed three times with PBS and stained with a solution containing 0.1% Triton-X100, 50µg/ml PI and 100µg/ml RNase for 15min at 37°C. The cells were analysed using flow cytometry (FACS Canto II, BD Biosciences, Germany) and quantified as percent cells in the respective cell cycle phase.

Tracking of lysosome stability

Labelling of lysosomes was performed as previously described in Messner et al.(Messner et al. 2012) Endothelial cells with or without inhibitor and p53 knock-down (KD) cells were incubated with 15µM and 30µM Cd respectively for the indicated times. As indicated, cells were pre-incubated with 50µM calpain inhibitor or 2mM 3MA for 30 min before Cd treatment.

Detection and quantification of cellular DNA content and PI staining.

After the treatment, endothelial cells were enzymatically detached. Following permeabilization of cell membranes with 1mg/ml saponin, the DNA was stained with PI and the DNA content was quantified by flow cytometry (FACS Canto II, BD Biosciences, Germany). Inhibitors used were: 50µM Aurintricarboxylic acid

ammonium salt (ATA; A0885, Sigma-Aldrich) and 1mM EGTA (ethylene glycol tetra-acetic acid, Sigma Aldrich). As indicated, cells were pre-incubated with 50µM calpain inhibitor or 2mM 3MA for 30 min before Cd treatment.

Generation of stable Bcl-XL OE cells as well as p53 KD cells.

Generation of stable BCL-XL OE and p53 KD endothelial cells was performed as already described. (Messner et al. 2012)

Western Blot analyses

Whole protein extracts from Cd treated and control cells were obtained by incubation of detached cells in triple detergent lysis buffer (50mM Tris-Chloride, 150mM Sodium Chloride, 0.02% Sodium Azide, 0.1% SDS, 1% Nonident P-40, 0.5% Sodium Deoxycholate, 5µg/ml Aprotinin, 1µg/ml Leupeptin, 1µg/ml Pepstatin and 1mM ABESF). Cells were lysed by repeated freezing and thawing and subsequent sonication. After a centrifugation step, the protein concentration of the supernatant was determined using a BCA protein assay kit (Pierce) according to the manufacturer's instructions. Equal amount of proteins were separated on SDS-polyacrylamide gels and subsequently transferred onto a nitrocellulose membrane (Schleicher and Schuell, Germany). The primary antibodies used were: mouse anti-p53 (#554294, BD, Austria), rabbit anti-BCL-XL (#2764, Cell Signaling, Germany) as well as rabbit anti-GAPDH (ab9485; Abcam, UK, dilution 1:2500) and the secondary antibodies used were goat anti-mouse IgG antibody-HRP conjugate (#32430, Thermo Scientific, Austria) and goat anti-rabbit IgG antibody-HRP conjugate (#31460, Thermo Scientific, Austria). To control equal protein amount loading, membranes were stained with Ponceau S. Furthermore equal loading was controlled by evaluating GAPDH expression.

RESULTS

Cd induced increase in intracellular Ca^{2+} concentration over time.

Ca^{2+} is an important intracellular messenger molecule and is known to be involved in the induction of cell death. Cd induces a significant increase in the intracellular Ca^{2+} concentration, which already starts 1h after Cd treatment and steadily increases over 48h. Figure S1 shows that Ca^{2+} concentration increased over time, whereby the increase depends on the Cd concentration. Treatment with a higher Cd concentration of 30µM provokes a stronger increase in intracellular Ca^{2+} concentration in comparison to the incubation with 15µM Cd.

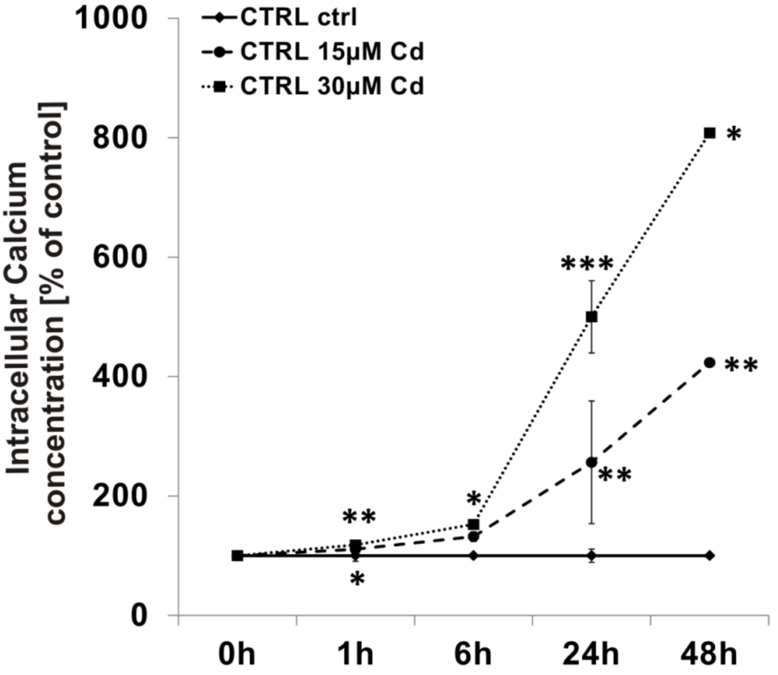

Figure S1: Treatment of endothelial cells with different Cd concentrations increases intracellular Ca^{2+} concentration over time. Figure S1 shows the flow cytometry-based quantification of intracellular Ca^{2+} concentration in wild type endothelial cells after treatment with 15µM Cd and 30µM Cd respectively for 48h. All experiments were performed in triplicates and were repeated at least three times. Results depict the mean ± standard deviation. Asterisks indicate significant differences compared with the corresponding control (* $p<0.05$; ** $p<0.01$, *** $p<0.001$).

Cd treatment does not change intracellular ATP concentration.

As apoptotic cell death is known to be an energy consuming process (in contrast to necrosis), we analysed the intracellular ATP concentration in endothelial cells after Cd treatment. Detailed analyses revealed that treatment with 15µM Cd initially induces a significant increase in intracellular ATP concentration after 6h, but a non-significant drop in the concentration over 72h. In contrast, treatment of cells with 30µM Cd causes no increase in intracellular ATP concentration but a significant decrease after 72h (Figure S2).

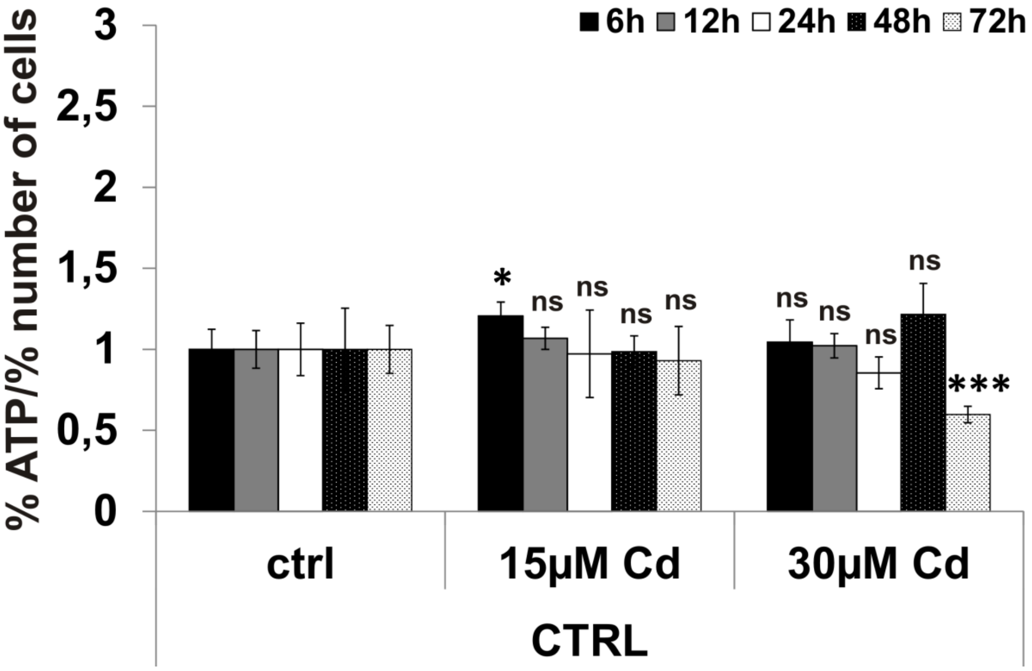

Figure S2: Intracellular ATP concentration of Cd treated endothelial cells. Endothelial cells were treated with 15µM and 30µM Cd respectively over a time period of 72h. Intracellular amount of ATP was quantified and analysed as percent per number of cells. All experiments were performed in quadruplicates and were repeated at least three times. Results depict the mean ± standard deviation. Asterisks indicate significant differences (* $p<0.05$; *** $p<0.001$) compared with the controls; ns = not significant.

Cd treatment inhibits cell proliferation and p53 KD is able to preserve cells from proliferation arrest.
Treatment of endothelial cells with 15µM Cd reduces the proliferation ability after 48h significantly, whereas incubation with 30µM Cd exerts its proliferation inhibiting effect already after 24h. Further on, untreated cells grow exponentially but Cd reduces the number of cells over 96h. Compared to wild type endothelial cells stable knock-down of p53 protects cells from Cd induced proliferation arrest significantly. However, this knock-down is not able to preserve the proliferation ability after Cd treatment entirely (Figure S3).

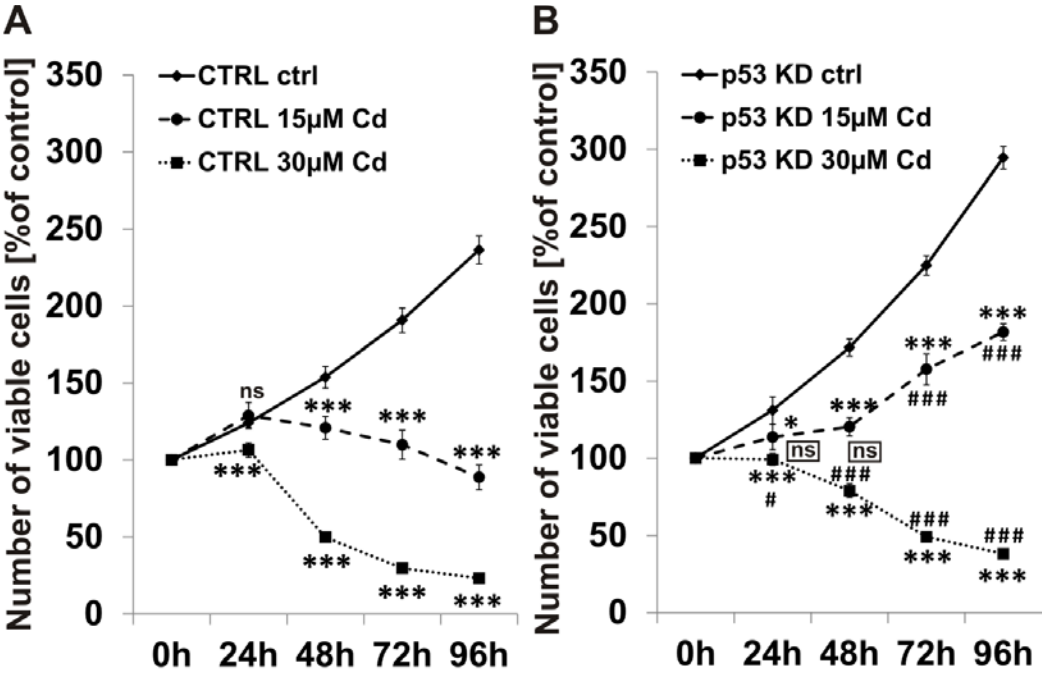

Figure S3 A and B: Analysis of the effect of Cd treatment on the proliferation of wild type endothelial cells and cells with stable p53 KD. Endothelial cells were treated with 15µM and 30µM Cd respectively over a time period of 96h. The number of viable cells relating to the proliferation ability of endothelial cells was analysed using an XTT-assay. Figure S3 A depicts the proliferation of wild type endothelial cells under Cd treatment over 96h, whereas Figure S3 B shows the proliferation of p53 KD cells under Cd treatment over 96h. All experiments were performed in quadruplicates and were repeated at least three times. Results depict the mean ± standard deviation. Asterisks indicate significant differences compared to the corresponding controls (* $p<0.05$; *** $p<0.001$; ns = not significant) and hash signs between CTRLs (HUVECs) and p53 KD cells (### $p<0.001$) ns = not significant.

In contrast, pre-treatment of endothelial cells with an inhibitor of calpain I and II is not as effective as a knock-down of p53 in influencing the Cd induced proliferation arrest, although the protective effect is significant after 96h of treatment. In summary, neither the knock down of p53 nor the inhibition of calpains is able to completely reverse the Cd induced proliferation arrest (Figure S4).

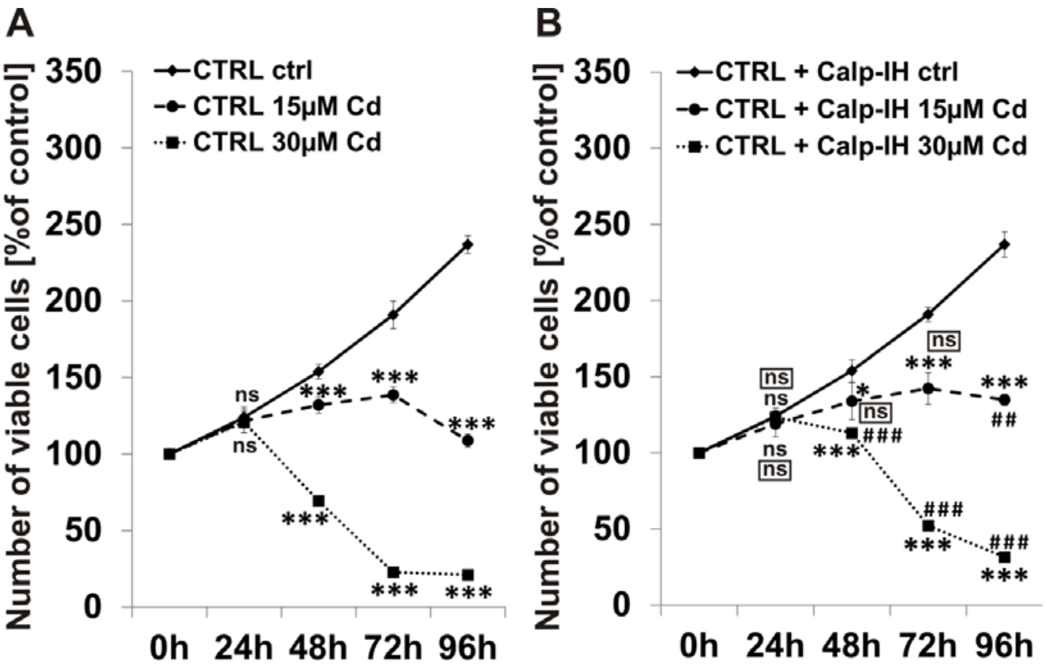

Figure S4 A and B: Analysis of the effect of Cd treatment on the proliferation of wild type endothelial cells and cells incubated with an inhibitor of calpain I and II. Endothelial cells were treated with 15µM and 30µM Cd respectively over a time period of 96h. The number of viable cells relating to the proliferation ability of endothelial cells was analysed using an XTT-assay. Figure S4 A shows the proliferation of CTRL cells treated with 15µM and 30µM Cd over 96h. Figure S4 B shows the proliferation of Cd treated cells pre-incubated with the calpain inhibitor over 96h. All experiments were performed in quadruplicates and were repeated at least three times. Results depict the mean ± standard deviation. Asterisks indicate significant differences compared to the corresponding controls (* $p<0.05$; *** $p<0.001$; ns = not significant) and hash signs between HUVECs (CTRL) and HUVECS pre-treated with an inhibitor of calpain I and II (### $p<0.001$) ns = not significant.

Cd treatment does not impair endothelial cell cycle

To further analyse Cd induced signalling pathways leading to endothelial cell death, we incubated endothelial cells with 15µM and 30µM Cd respectively for 6h. After treatment the cells were subjected to flow cytometry-based cell cycle analyses. As shown in Figure S5, Cd treatment does not influence the endothelial cell cycle.

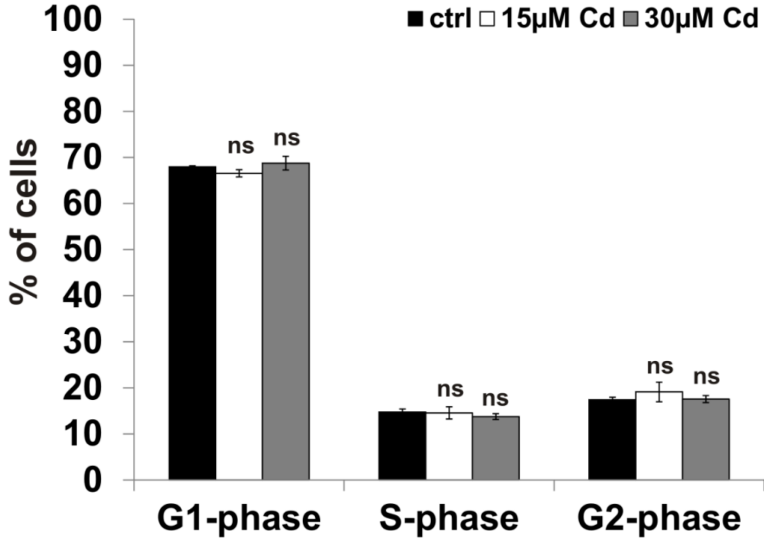

Figure S5: Influence of Cd treatment on endothelial cell cycle. Endothelial cells were treated with 15µM and 30µM Cd respectively. After 6h of treatment, endothelial cells were subjected to flow cytometry-based cell cycle analyses. Figure S5 shows, that Cd incubation does not inhibit endothelial cell cycle. All experiments were performed in quadruplicates and were repeated at least three times. Results depict the mean ± standard deviation (ns = not significant).

Cd induced lysosome permeabilization is neither preventable by the inhibition of calpains nor by the inhibition of autophagy by 3MA.

Cd treatment induces a massive disruption of lysosomes in endothelial cells. Although the inhibition of calpains and autophagy is able to inhibit Cd induced cell death, flow cytometry-based analyses showed that neither inhibition of calpain activity (Figure S6 A) nor inhibition of autophagy by 3MA (Figure S6 B inhibits lysosomal breakdown.

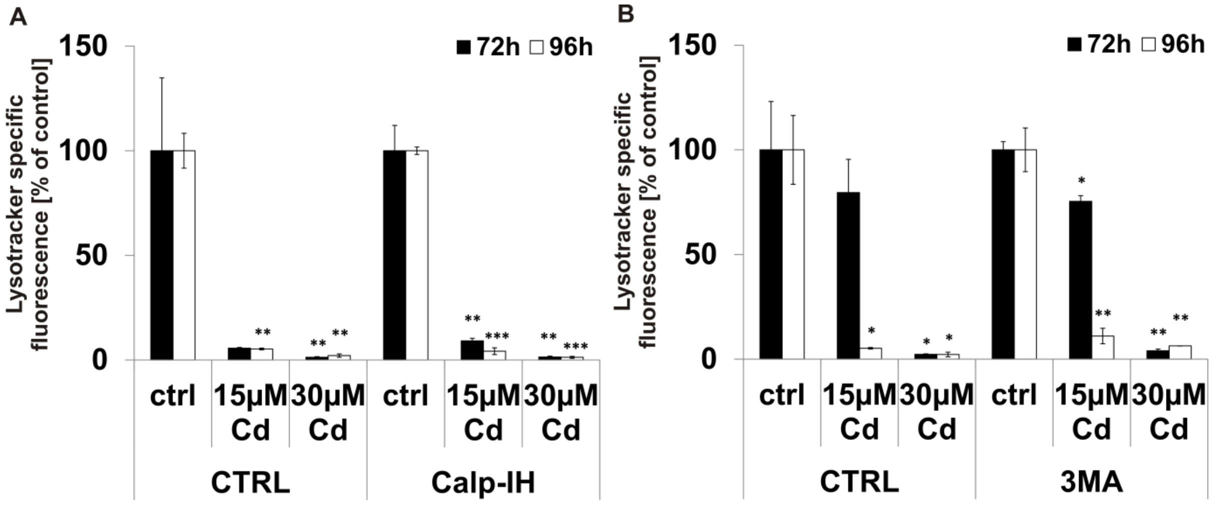

Figure S6 A and B: Neither the inhibition of calpain activity, nor the inhibition of autophagy prevents Cd induced lysosomal breakdown. Endothelial cells were incubated with 50µM

calpain inhibitor or 2mM 3MA prior to Cd treatment for 72h and 96h respectively. Lysosomes were stained with Lysotracker and the integrity of these organelles was quantified using flow cytometry based-analyses. Figure S6 A shows that the inhibition of calpain activity was not effective in the prevention of lysosomal breakdown. Figure S6 B depicts that the inhibition of autophagosome formation by 3MA was not effective in preventing lysosomal breakdown. All experiments were performed in quadruplicates and were repeated at least three times. Results depict the mean ± standard deviation. Asterisks indicate significant differences compared to the corresponding controls (* $p<0.05$; ** $p<0.01$; *** $p<0.001$).

Cd induced DNA degradation is neither preventable by calpain inhibitor nor by 3MA incubation.

Cd induced cell death is characterized by a distinct DNA degradation in the course of cell death signalling. As depicted in Figure S7 A, inhibition of calpain activity was not able to inhibit DNA degradation; on the contrary, inhibition of calpain activity accelerates DNA degradation. Similar results were obtained by the analyses of DNA content in cells incubated with 3MA prior to Cd treatment. Figure S7 B shows that inhibition of 3MA was not able to prevent DNA degradation, also this treatment seems to accelerate DNA degradation significantly.

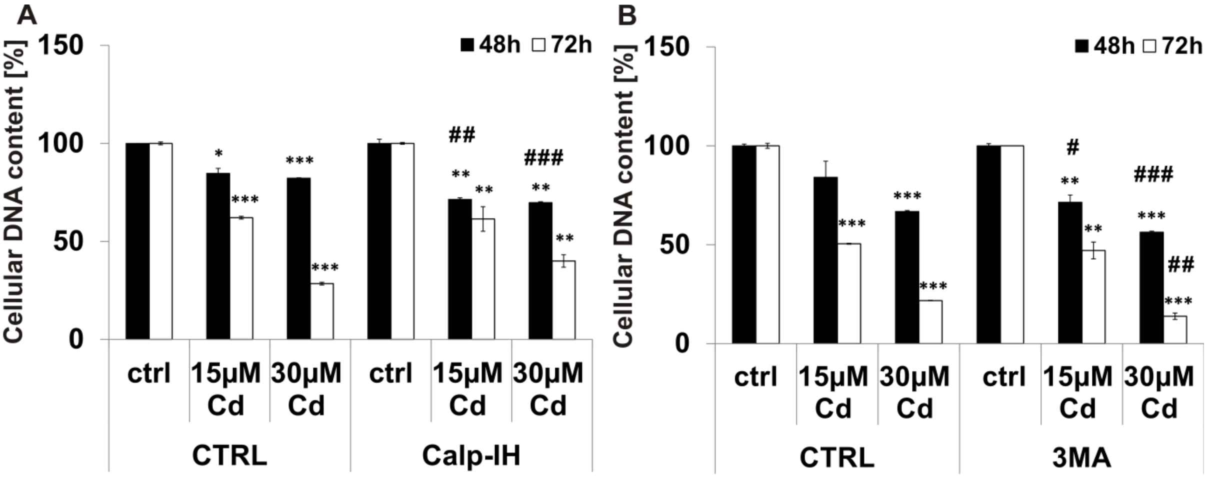

Figure S7 A and B: Influence of calpain inhibition and 3MA incubation on Cd induced DNA degradation. Endothelial cells were pre-incubated with the two indicated inhibitors for 30 min before Cd treatment. Figure S7 A shows inhibition of calpain activity was not able to prevent DNA degradation, on the contrary a significant acceleration of DNA degradation could be observed. Likewise, pre-incubation of Cd treated cells with 3MA showed no preventive effect, but rather an enhancement of Cd induced DNA degradation (Figure S7 B). All experiments were performed in quadruplicates and were repeated at least three times. Results depict the mean ± standard deviation. Asterisks indicate significant differences compared to the corresponding controls (* $p<0.05$; ** $p<0.01$; *** $p<0.001$) and hash signs indicate significant differences between the groups (# $p<0.05$; ## $p<0.01$; ### $p<0.001$).

Cd induced DNA degradation is preventable by EGTA treatment, but not by ATA treatment.

Pre-treatment of Cd treated cells with the Ca^{2+}-Mg^{2+}- dependent endonuclease inhibitor Aurintricarboxylic Acid (ATA) does not abolish the progressive DNA degradation (in contrast significant reduction of the DNA amount

by ATA in the 30µM Cd group compared to the corresponding control can be observed; Figure S8 A). As was to be expected, incubation of Cd treated endothelial cells with the chelating agent EGTA (ethylene glycol tetraacetic acid) completely protects cells from DNA degradation (Figure S8 B).

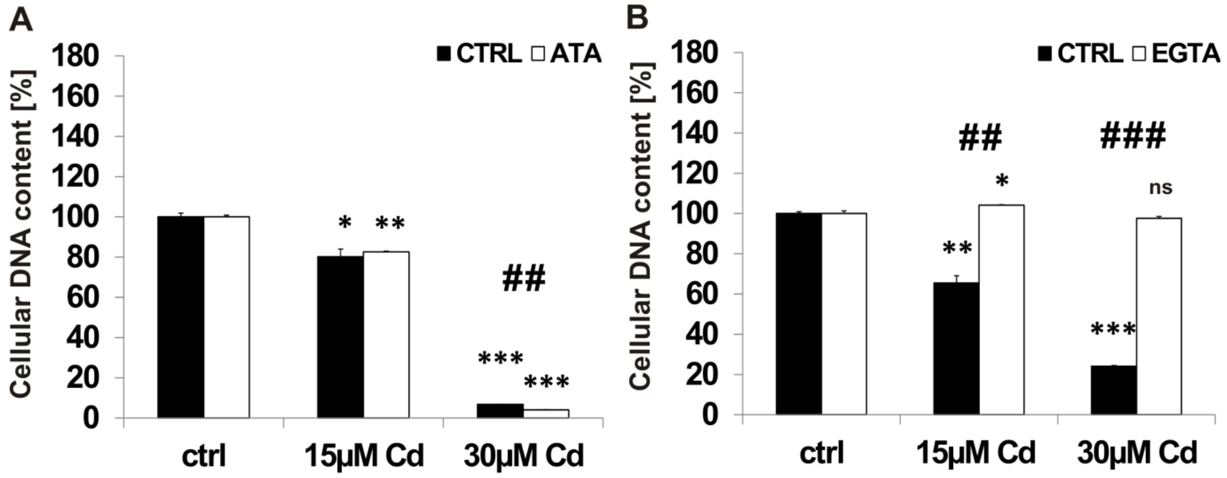

Figure S8 A and B: Influence of EGTA and ATA pre-incubation on the DNA content of Cd treated endothelial cells. Cells were pre-incubated either with 50µM ATA or 1mM EGTA for 30min and then subjected to Cd treatment. After 96h of treatment, DNA content was quantified by Flow cytometry-based analyses. Figure S8 A shows that ATA pre-incubation was not able to prevent Cd-induced DNA degradation, whereas EGTA pre-incubation completely protects Cd treated endothelial cells from DNA degradation (Figure S8 B). Results depict the mean ± standard deviation. Asterisks indicate significant differences compared to the corresponding controls (* $p<0.05$; ** $p<0.01$; *** $p<0.001$; ns = not significant) and hash signs between endothelial cells without and with inhibitor incubation (## $p<0.01$; ### $p<0.001$); ns= not significant.

Supplementation of Cd treated cells with energy suppliers like Fructose, Sucrose, and Mannitol does not rescue cells from death effectively.

As apoptosis is known to be an energy dependent process and autophagy is known to be induced by nutrient starvation, we incubated Cd treated endothelial cells with energy suppliers. As depicted in Figure S9 A, incubation of Cd treated cells with various Fructose concentrations was not able to completely inhibit cell death, although incubation with 20mM Fructose significantly reduces the amount of dead cells induced by treatment with 15µM Cd. In summary, no biological relevant trend towards a reduction of cell death following Fructose incubation could be observed. Likewise, the incubation of Cd-treated endothelial cells with increasing Sucrose concentrations does not rescue cells completely from death. However, Supplemental Figure IX B shows that Sucrose incubation reduces the amount of dead cells in a concentration dependent manner. Although not all comparisons revealed significant results, Figure S9 B depicts a concentration dependent biological trend towards a protective effect of Sucrose. The third energy supplier used to inhibit Cd induced cell death was Mannitol. Incubation of Cd treated endothelial cells revealed similar results as Sucrose. As depicted in Figure S9 C, incubation with Mannitol partially inhibits Cd-induced cell death and a concentration dependent biological trend towards the reduction in the amount of cells could also be seen in this incubation schema.

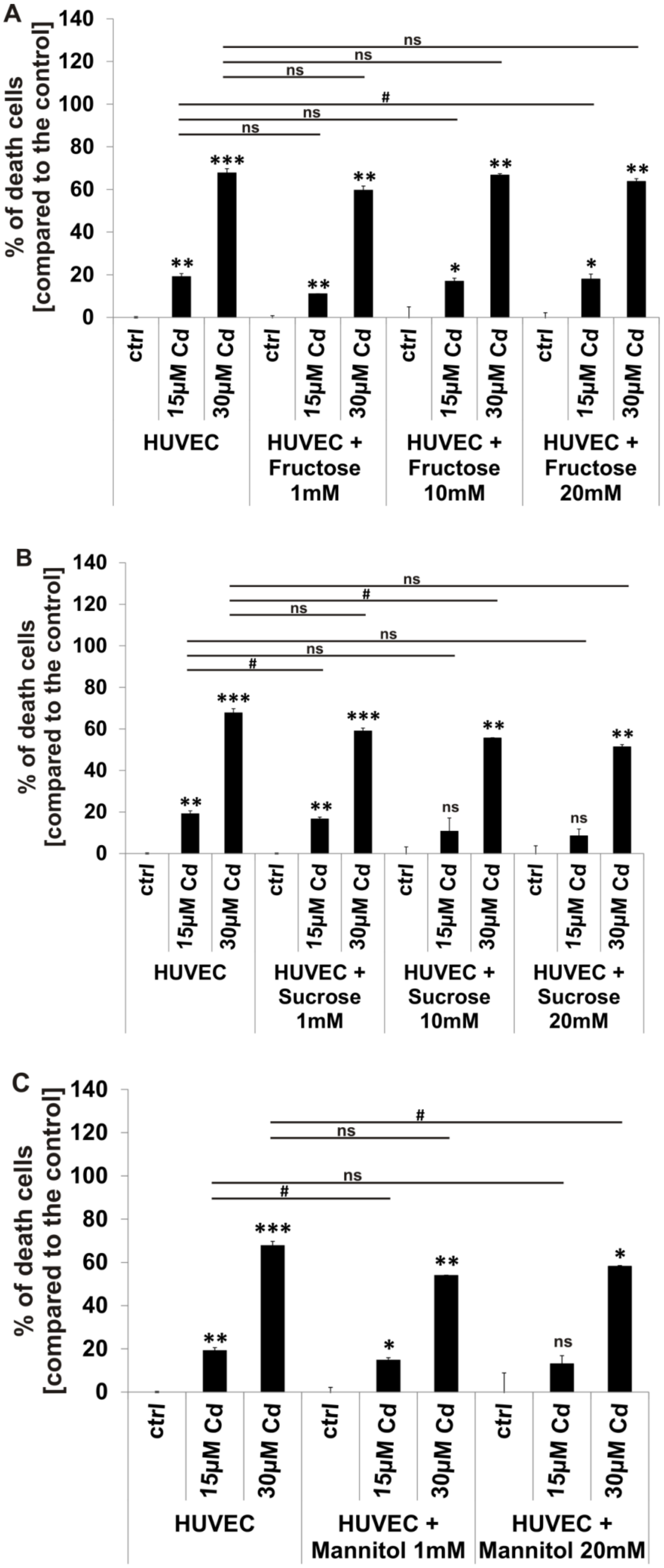
A
B
C
% of death cells
[compared to the control]
140
120
100
80
60
40
20
0
ns
#
*
**
***
ctrl
15µM Cd
30µM Cd
HUVEC
HUVEC + Fructose 1mM
HUVEC + Fructose 10mM
HUVEC + Fructose 20mM
HUVEC + Sucrose 1mM
HUVEC + Sucrose 10mM
HUVEC + Sucrose 20mM
HUVEC + Mannitol 1mM
HUVEC + Mannitol 20mM

Figure S9 A-C: Rescue of Cd treated endothelial cells from cell death by incubation with Fructose, Sucrose and Mannitol. Endothelial cells were treated with 15µM and 30µM Cd respectively and simultaneously incubated with increasing concentrations of Fructose, Sucrose, and Mannitol for 96h. Figure S9 A shows that Fructose was not able to inhibit Cd-induced cell death significantly. In contrast, incubation of Cd-treated cells with Sucrose or Mannitol significantly reduces the amount of dead cells (Figure S9 B and C). Results depict the mean ± standard deviation. Asterisks indicate significant differences compared to the corresponding controls (* $p<0.05$; ** $p<0.01$; *** $p<0.001$; ns = not significant) and hash signs between CTRL and CTRL pre-treated with an inhibitor of calpain I and II (# $p<0.05$) ns = not significant.

Monitoring of stable p53 KD as well as BCL-XL OE in endothelial cells.

Protein extracts of CTRL cells (wild type endothelial cells) and p53 KD cells were prepared and equal amounts of protein were loaded. Western blot based detection of the expression of p53 was performed. Figure S10 shows that the expression of p53 is strongly reduced in the lentiviral transfected cells compared to wild type endothelial cells. Detection of GAPDH loading control revealed an increased loading of proteins in the p53 KD lane compared to the CTRL lane. Nevertheless, calculation of expression ratios between p53 and GAPDH showed the obvious KD of p53 (ratio 1.91 in CTRL compared to 0.59 in p53 KD cells).

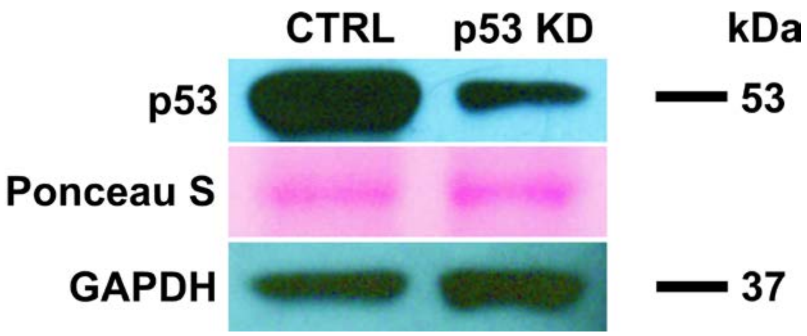

Figure S10: Western Blot based analyses of the expression level of p53 in CTRL and p53 KD cells. Equal amounts of protein extract form CTRL as well as p53 KD cells were separated, blotted onto a nitrocellulose membrane, and p53 protein was detected. Figure S10 shows that p53 was knocked down in the lentiviral transfected cells compared to the wild type endothelial cells.

Protein extracts of CTRL cells (wild type endothelial cells) and BCL-XL OE cells were prepared and equal protein amounts were loaded. Western blot based detection of the expression of BCL-XL was performed. Figure S11 shows that the expression BCL-XL is strongly over expressed in the lentiviral transfected cells compared to wild type endothelial cells. Detection of GAPDH loading control revealed a strongly increased loading of proteins in the BCL-XL OE lane compared to the CTRL lane. Nevertheless, calculation of expression ratios between BCL-XL and GAPDH showed the obvious OE of BCL-XL (ratio 0.019 in CTRL compared to 0.72 in BCL-XL OE cells).

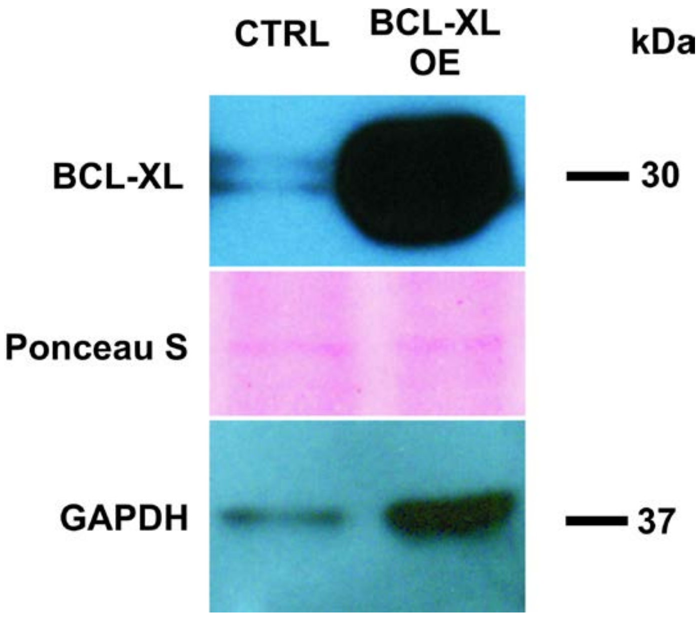

Figure S11: Western Blot based analyses of the expression level of BCL-XL in CTRL and BCL-XL OE cells. Equal amounts of protein extract form CTRL as well as BCL-XL OE cells were separated, blotted onto a nitrocellulose membrane, and BCL-XL protein was detected. Figure S11 shows that BCL-XL was over-expressed in the lentiviral transfected cells compared to the wild type endothelial cells.

REFERNCES

Bernhard D, Pfister G, Huck CW, Kind M, Salvenmoser W, Bonn GK, et al. 2003. Disruption of vascular endothelial homeostasis by tobacco smoke: Impact on atherosclerosis. FASEB journal : official publication of the Federation of American Societies for Experimental Biology 17:2302-2304.

Messner B, Ploner C, Laufer G, Bernhard D. 2012. Cadmium activates a programmed, lysosomal membrane permeabilization-dependent necrosis pathway. Toxicology letters 212:268-275.
